# Identifying Mild Cognitive Impairment from Routinely Collected Physical, Functional and Psychosocial Measures in Community-Dwelling Older Adults: A Comparative Machine-Learning and Regression Modelling Study in the Northern Japanese ORANGE Registry

**DOI:** 10.3390/geriatrics11050138

**Published:** 2026-10-01

**Authors:** Ayuto Kodama, Takako Ohnuma, Kana Sasaki, Kaoru Sugawara, Nobuhiro Fujiyama, Youko Umetsu, Tsuyoshi Ono, Hidetaka Ota

**Affiliations:** 1 Department of Occupational Therapy, Graduate School of Medicine, Akita University, 1-1-1 Hondo, Akita 010-8543, Japan; ay-kodama@med.akita-u.ac.jp; 2 Advanced Research Center for Geriatric and Gerontology, Akita University, 1-1-1 Hondo, Akita 010-8543, Japan; ohnuma@med.akita-u.ac.jp (T.O.); s4005524sk@gmail.com (K.S.); kaoruko02@jimu.akita-u.ac.jp (K.S.); 3 Clinical Research Promotion and Support Office, Future Cooperative Research Organization, Akita University, 1-1-1 Hondo, Akita 010-8543, Japan; fujiyama@hos.akita-u.ac.jp; 4 Integrated Community Support Center, Public Health and Welfare Department, City Hall of Yokote, Yokote, Akita 010-8543, Japan; umetsu-yoko-a@city.yokote.lg.jp; 5 Omori Municipal Hospital, Yokote, Akita 010-8543, Japan; oulan1915@yahoo.co.jp

**Keywords:** mild cognitive impairment, machine learning, logistic regression, clinical prediction model, community-dwelling older adults, calibration, decision-curve analysis

## Abstract

**Background**: Community health check-ups routinely collect non-cognitive data. Whether these support mild cognitive impairment (MCI) triage, and whether machine learning outperforms regression, is unclear. **Methods**: Cross-sectional analysis of 2650 community-dwelling adults aged ≥ 65 in Akita, Japan (2018–2025). MCI was defined by the NCGG-FAT (1.5 SD, age/education-adjusted norms). Thirty-four pre-specified non-cognitive predictors were modelled using tree ensembles, penalised regression, other machine-learning families and simple reference scores, with stratified 70/30 splitting, DeLong tests, 100 repeated splits, training-derived thresholds, calibration metrics and decision-curve analysis. **Results**: MCI prevalence was 28.6%. Random forest discriminated best (ROC AUC 0.722, 95% CI 0.681–0.761; PR-AUC 0.542) and was well calibrated (slope 1.048; intercept −0.024), but its advantage over LASSO regression was marginal (ΔAUC +0.031, *p* = 0.044) and absent versus gradient boosting (*p* = 0.059). All multivariable models greatly outperformed the Kihon Checklist (AUC 0.550, *p* < 0.001). Neither operating point was deployable: at 0.5, sensitivity was 0.269 (166/227 cases missed); at the training-derived Youden threshold (0.321), sensitivity was 0.604, specificity 0.699, and PPV 0.445. Net benefit exceeded assess-all/assess-none between threshold probabilities 0.15–0.60. **Conclusions**: Routine non-cognitive data support moderate, well-calibrated MCI discrimination, but machine learning added little over regression. This is an exploratory triage aid requiring external validation.

## 1. Introduction

Mild cognitive impairment (MCI) is an intermediate clinical state between normal cognitive ageing and dementia, associated with elevated risk of progression and functional decline [1]. Dementia prevention has become a major public health priority, as emphasised in successive Lancet Commission reports advocating multidomain risk-reduction strategies [2,3], and diagnostic frameworks increasingly emphasise biological and clinical staging across the cognitive continuum [4]. Despite these advances, population-level screening remains challenging. The U.S. Preventive Services Task Force has noted uncertainty regarding the net benefit of universal cognitive screening in asymptomatic older adults, reinforcing the need for pragmatic, scalable approaches to identify individuals who may benefit from further assessment [5].

A growing body of work has applied machine learning (ML) to community-based, non-imaging data for MCI identification, but reported performance has been heterogeneous and generally modest. In the Korean Longitudinal Study of Ageing (*n* = 4975), a systematic comparison of nine algorithms found that the best-performing model for incident MCI was a random forest with a median area under the receiver operating characteristic curve (AUC) of 0.673, and the authors concluded that no model achieved robust accuracy for MCI [6]. In a Chinese community cohort (*n* = 1503), a nomogram combining conventional risk factors with Traditional Chinese Medicine constitution types reached an AUC of 0.813 in training and 0.747 in internal validation at an MCI prevalence of 24.1% [7]. Studies that incorporate detailed neuropsychological testing or neuroimaging achieve substantially higher discrimination [8], but such data are not available in routine community health check-ups. Three limitations recur in this literature and motivate the present study. First, tree-based ensembles are frequently benchmarked only against one another, so the incremental value of nonlinear modelling over penalised regression is rarely established. Second, performance is often reported from a single random train/test split without repeated resampling, so the ranking of algorithms may reflect split-specific noise. Third, decision thresholds are sometimes optimised on the same data used for evaluation, calibration is reported qualitatively if at all, and clinical utility is asserted rather than quantified. We therefore designed this study explicitly to test whether machine learning adds measurable value over conventional statistical modelling in this setting, rather than to demonstrate that it does.

Predictors were restricted to non-cognitive variables recorded at the same health check-up visit, to avoid outcome leakage. The intended use of such a model is pre-screening triage: within a municipal health check-up programme in which cognitive testing capacity is limited, the model would run on data already collected at the visit and prioritise which attendees are invited for formal cognitive assessment. It is not intended to replace cognitive testing, to be used after cognitive testing has been performed, or to support a diagnosis. Under this intended use, the relevant performance question is not overall accuracy but whether the model can concentrate MCI cases into a manageable referral pool at an acceptable rate of missed cases—which is why we report sensitivity, positive predictive value, calibration and net benefit rather than accuracy alone. We aimed to (i) develop and internally evaluate models classifying MCI from routinely collected demographic, medical, physical and psychosocial data; (ii) compare tree-based ML against regularised regression, other ML families and simple reference scores, with formal statistical testing of AUC differences and assessment of stability across resampling; (iii) quantify calibration and clinical utility; and (iv) identify influential predictors and derive interpretable representations, including an exploratory fuzzy rule base, for clinical communication.

## 2. Materials and Methods

### 2.1. Study Design, Setting and Participants

This cross-sectional modelling study used data from the ORANGE Registry community health check-up programme conducted in Akita Prefecture, Japan, between 2018 and 2025. Check-ups were held at municipal community venues (principally Yokote Hall) and at Omori Municipal Hospital.

Sampling and recruitment. Recruitment was by open invitation rather than probability sampling. Participating municipalities publicised the check-up through municipal newsletters, community-centre notices, neighbourhood associations and direct mailing to residents aged 65 years or older on the resident register; attendance was voluntary and free of charge. All attendees who met the eligibility criteria and provided written informed consent were enrolled consecutively. Because participation was voluntary, the sample is best characterised as a self-selected community volunteer sample, and this is addressed in Section 4.6.

Eligibility. Inclusion criteria were age 65 years or older, community-dwelling status, and ability to walk independently and live at home without personal assistance. Exclusion criteria were dementia, major depression, severe hearing or visual impairment, stroke, Parkinson’s disease, other neurological disease, intellectual disability, and certified need for support or care under the Japanese public long-term care insurance system owing to disability.

Participant flow. The registry database was assembled from participants enrolled after eligibility screening. Consequently, the number of residents invited, the number attending each check-up and the number excluded under each criterion were not retained in the programme records and cannot be reported; the flow of participants from enrolment to the analytic sample and the training/test split is shown in Supplementary Figure S1. We acknowledge this as a reporting limitation.

Ethics. The study was approved by the Ethics Committee of the Faculty of Medicine, Akita University (Approval No. 273; approved 1 October 2017) and conducted in accordance with the Declaration of Helsinki. Written informed consent was obtained from all participants.

### 2.2. Outcome Definition

The outcome was binary MCI status (0 = non-MCI, 1 = MCI). Following Petersen’s criteria [9], MCI was defined as objective cognitive impairment without loss of independence in activities of daily living, operationalised using the National Center for Geriatrics and Gerontology Functional Assessment Tool (NCGG-FAT).

The NCGG-FAT battery. The NCGG-FAT is a computerised battery administered on a tablet computer ([manufacturer, city, country]) developed and validated for community-based assessment in Japanese older adults [10]. It comprises subtests covering four cognitive domains: (i) memory—word-list immediate recall and word-list delayed recall (10 words, three learning trials, free recall after a delay); (ii) attention and executive function—Trail Making Test parts A and B, administered on the tablet with automated timing; (iii) processing speed—Symbol Digit Substitution Test (number of correct substitutions in 90 s); and (iv) working memory—forward and backward digit span. Each subtest is scored automatically, removing examiner-dependent variability. Total administration time is approximately 20 min. The battery has demonstrated acceptable test–retest reliability and concurrent validity against conventional paper-and-pencil neuropsychological tests [10].

Norms and cut-off. Raw subtest scores were converted to standardised scores using age- and education-adjusted normative data derived from large Japanese community-dwelling cohorts assembled by the National Center for Geriatrics and Gerontology [10,11]. Impairment in a given domain was defined as performance more than 1.5 SD below the age- and education-adjusted normative mean.

Justification of the 1.5 SD cut-off. The 1.5 SD threshold is the criterion originally proposed in the Petersen operationalisation of MCI [9] and is the criterion under which the NCGG-FAT normative database was constructed and validated [10,11]. A 5-year prospective study using this operationalisation reported that NCGG-FAT-defined cognitive states predicted incident dementia with hazard ratios of 1.55–2.92 for MCI subtypes, supporting the prognostic validity of the 1.5 SD definition in this population [11]. A stricter 2.0 SD cut-off would identify a smaller, more severely impaired group and is less suited to a preventive, community-screening context, whereas a 1.0 SD cut-off substantially inflates prevalence and lowers specificity for progression. We therefore retained 1.5 SD for comparability with the normative database and with the broader Japanese literature. Because the choice of cut-off directly determines outcome prevalence and hence model performance, this is acknowledged as a source of outcome-definition dependence in Section 4.6.

### 2.3. Candidate Predictors and Rationale for Selection

Candidate predictors were restricted, by design, to non-cognitive variables collected at the same visit; no NCGG-FAT scores or other cognitive test results were used as predictors. Within that constraint, the predictor set was pre-specified before modelling on the basis of three considerations: (a) the variable is routinely collected in Japanese municipal health check-ups and would therefore be available at the point of use without additional measurement burden; (b) there is prior evidence linking the domain to cognitive decline; and (c) the variable is measured identically across participating municipalities. No data-driven pre-screening of predictors (for example, univariable filtering by *p*-value) was performed, as this would bias downstream performance estimates.

The pre-specified domains and their rationale were: demographics (age, sex, years of education—established determinants of cognitive reserve and MCI risk); comorbidity (hypertension, stroke, cardiac disease, diabetes, hyperlipidaemia, osteoporosis, respiratory disease, knee osteoarthritis, fracture after age 60, malignant neoplasm, Parkinson’s disease, physician-diagnosed depression, rheumatoid arthritis/collagen disease, and number of daily medications—vascular, metabolic and inflammatory pathways, plus polypharmacy as a marker of multimorbidity); physical function (usual gait speed, grip strength measured with a Smedley-type handgrip dynamometer ([manufacturer, city, country]), height, body weight, ≥5% weight loss over two years, physical frailty status by the Fried phenotype [12] in its Japanese NCGG-SGS operationalisation [13]); psychosocial factors (15-item Geriatric Depression Scale [GDS-15], social frailty status by the Makizako Social Frailty Index [14], living alone, number of household members, reduced daily conversation); and lifestyle (smoking status, light and regular exercise habit, Kihon Checklist total score, self-reported memory complaint). The complete predictor list with definitions, coding and measurement procedures is given in Supplementary Table S2.

Handling of neurological and psychiatric diagnoses. Reviewers of the initial submission correctly noted that including diagnoses lying on the cognitive-decline spectrum requires justification in a community-screening model. On inspection of the assembled dataset, Alzheimer’s disease had zero recorded cases (0/2650) and therefore carried no information; it was removed as a zero-variance predictor. Parkinson’s disease (1 case) and physician-diagnosed depression (7 cases) were extremely rare, a direct consequence of the exclusion criteria, which removed participants with dementia, Parkinson’s disease, other neurological disease, major depression and stroke before enrolment. The near-absence of these diagnoses is therefore a design feature rather than a chance finding, and it is the reason why Alzheimer’s disease carried no information at all. These variables were retained in the primary model for completeness of the pre-specified set, but a pre-planned sensitivity analysis excluding all neurological and psychiatric diagnoses (Parkinson’s disease, depression, self-reported stroke) was performed to confirm that the model does not depend on near-outcome conditions (Section 3.9). A second sensitivity analysis excluded self-reported memory complaint, which, although not a cognitive test, could be argued to lie close to the outcome.

Multicollinearity. Pairwise Spearman correlations and variance inflation factors (VIFs) were computed for all candidate predictors on the complete dataset (Supplementary Table S5).

### 2.4. Data Quality Control, Splitting and Preprocessing

Prior to analysis, each variable was screened against a pre-specified plausible range, and values outside that range were treated as missing rather than imputed as recorded (for example, recorded ages below 60 or above 110 years, heights outside 120–200 cm, GDS-15 scores above 15). This affected 139 of 90,100 cells (0.15%). The plausible ranges are listed in Supplementary Table S2.

Data were then stratified by outcome and randomly split into training (70%, *n* = 1855) and test (30%, *n* = 795) sets. Preprocessing comprised median imputation of missing numeric values, removal of zero-variance predictors, and z-score standardisation. All preprocessing parameters (medians, means, SDs, zero-variance flags) were estimated on the training data only and applied unchanged to the held-out test set. Although tree-based models are largely scale-invariant, standardisation was applied to maintain an identical pipeline across all model families, several of which (support vector machine, k-nearest neighbours, neural network, penalised regression) are scale-sensitive.

### 2.5. Models Compared

To address whether the complexity of nonlinear ensemble modelling is warranted, and to avoid restricting the comparison to a single model family, we fitted the following:

Tree-based models: decision tree (CART), random forest, and gradient-boosted trees (XGBoost).

Regression-based models: unpenalised logistic regression; ridge (L2), LASSO (L1) and elastic-net penalised logistic regression, with the penalty parameter (and L1 ratio) tuned by cross-validation.

Other machine-learning families: support vector machine with radial basis kernel, k-nearest neighbours, Gaussian naïve Bayes, and a feed-forward neural network (multilayer perceptron).

Class-imbalance-aware variants: random forest with balanced sub-sampling class weights, and cost-sensitive XGBoost with ’scale_pos_weight’ set to the negative-to-positive class ratio.

Simple reference scores, representing current practice: age alone; the Kihon Checklist total score alone (the instrument currently used for frailty screening in Japanese municipalities); and a simplified seven-variable logistic score (age, education, GDS-15, gait speed, grip strength, physical frailty, social frailty) representing what could be computed by hand at a check-up desk.

Exploratory fuzzy inference system (Section 2.10).

All hyperparameters were tuned using stratified five-fold cross-validation within the training set with ROC AUC as the selection criterion. Final tuned values are reported in Supplementary Table S1.

### 2.6. Performance Evaluation and Statistical Comparison

Final models were refitted on the full training set and evaluated on the untouched test set. Reported metrics were ROC AUC, precision–recall AUC (PR-AUC, more informative than ROC AUC under class imbalance), Brier score, accuracy, sensitivity, specificity, positive and negative predictive value, F1 score and balanced accuracy, with MCI as the positive class. Confidence intervals for AUC were obtained by stratified bootstrap resampling (2000 replicates); intervals for classification metrics used Wilson score intervals.

Differences in AUC between the best model and every comparator were tested using the DeLong method for correlated ROC curves [15], with the difference and its 95% confidence interval reported alongside the *p* value. No adjustment for multiplicity was applied; comparisons are interpreted as exploratory and the direction of the conclusion does not rest on any single test.

### 2.7. Stability of Performance Estimates

Because a single 70/30 split yields an unstable estimate and can favour a particular algorithm by chance, we repeated the entire pipeline (split, preprocessing, fitting, evaluation) across 100 independent stratified random splits, and separately performed 5 × 5 repeated stratified cross-validation on the full dataset. We report the mean, SD and range of AUC for each model, the proportion of splits in which each model ranked first, and paired within-split differences between models.

### 2.8. Threshold Derivation and Class Imbalance

To ensure that the test set remained an untouched evaluation set, all decision thresholds were derived exclusively from training data, using out-of-fold predicted probabilities from the five-fold cross-validation within the training set. Two thresholds were derived: the Youden-index-maximising threshold, and the lowest threshold achieving ≥90% sensitivity in training (a screening-oriented operating point). These training-derived thresholds were then applied unchanged to the test set. For transparency, and because the initial submission of this manuscript reported a threshold derived from the test ROC curve, we additionally report the test-derived Youden threshold so that the magnitude of the resulting optimism can be seen directly.

Class imbalance (28.6% MCI) was addressed in three complementary ways: by reporting threshold-independent and imbalance-sensitive metrics (PR-AUC, balanced accuracy) rather than relying on accuracy; by fitting explicitly class-weighted and cost-sensitive variants of the two best models; and by evaluating screening-oriented operating points rather than the default 0.5 threshold, which is arbitrary when the outcome is not balanced.

### 2.9. Calibration and Clinical Utility

Calibration was assessed both graphically (calibration plot with locally estimated smoothing) and quantitatively. We report the Brier score, the calibration slope and calibration intercept from a logistic recalibration model regressing observed outcome on the logit of predicted probability, and calibration-in-the-large (the intercept with slope constrained to 1). Confidence intervals were obtained by bootstrap resampling (1000 replicates).

Clinical utility was assessed by decision-curve analysis [16], computing net benefit across threshold probabilities of 0.05–0.60 and comparing the models against the strategies of assessing all attendees and assessing none.

### 2.10. Exploratory Fuzzy Rule-Based Model

Following a reviewer’s suggestion that fuzzy modelling may provide a more general and more linguistically interpretable modelling platform, we additionally constructed a Mamdani-type fuzzy inference system. Six linguistic variables (age, years of education, GDS-15 score, usual gait speed, physical frailty status, social frailty status) were each partitioned into three Gaussian membership functions (low, medium, high) with centres placed at training-set quantiles. Fuzzy rules were generated from training data by the Wang–Mendel procedure [17]: each training record was assigned to the rule antecedent formed by its maximum-membership fuzzy set in each variable, the rule consequent was set to the membership-weighted mean outcome among records matching that antecedent, and rules supported by fewer than five records were discarded. Inference used product t-norm aggregation with weighted-average defuzzification. Partitions of three, four and five fuzzy sets per variable were compared. This model is reported as an exploratory, interpretability-oriented analysis and not as a competitor for the primary performance comparison.

### 2.11. Interpretation, Reporting and Risk of Bias

Permutation importance (mean decrease in test-set ROC AUC after permuting each predictor, 50 repetitions, with 2.5th–97.5th percentile intervals) was computed for the best-performing model. A shallow decision tree was fitted to the top-ranked predictors as a surrogate for clinical communication.

Reporting followed the TRIPOD+AI statement [18]. Risk of bias and applicability were assessed independently by two authors (A.K., H.O.) using PROBAST+AI [19], with disagreement resolved by discussion; the completed assessment is reported in full in Section 3.10. Considerations for early-stage clinical evaluation were informed by DECIDE-AI [20].

Analyses were conducted in R version 4.5.2 (R Foundation for Statistical Computing, Vienna, Austria) using the tidymodels framework (ranger version 0.17.0, rpart version 4.1.24, xgboost version 3.1.2.1, glmnet version 4.1-10), with cross-checking in Python ver-sion 3.12.3 (Python Software Foundation, Wilmington, DE, USA) using scikit-learn version 1.8.0 and xgboost version 3.4.1. Software versions are also listed in Supplementary Table S1. Analysis code is available from the corresponding author on reasonable request.

## 3. Results

### 3.1. Participants

The analytic sample comprised 2650 community-dwelling older adults (non-MCI *n* = 1893; MCI *n* = 757). Overall MCI prevalence was 28.6%. Stratified splitting yielded 1855 participants in the training set (MCI *n* = 530, 28.6%) and 795 in the test set (MCI *n* = 227, 28.5%).

Characteristics of participants with and without MCI are shown in Table 1. Participants with MCI were older (78.0 ± 6.3 vs. 75.4 ± 5.9 years; standardised mean difference [SMD] 0.425), had fewer years of education (11.5 ± 2.3 vs. 12.2 ± 2.2; SMD −0.326), slower usual gait speed (1.2 ± 0.3 vs. 1.3 ± 0.3 m/s; SMD −0.591), weaker grip strength, higher physical frailty status (SMD 0.336), higher Kihon Checklist scores and higher GDS-15 scores. Differences in individual comorbidities were small (all |SMD| < 0.16). Missing data ranged from 2.3% (age) to 31.5% (physician-diagnosed depression); variables with the highest missingness were those added later in the check-up protocol.

### 3.2. Multicollinearity Among Candidate Predictors

The maximum variance inflation factor across the 34 retained predictors was 2.95 (height), and no predictor had a VIF ≥ 5. Only one predictor pair exceeded |Spearman ρ| = 0.7 (number of household members and living alone, ρ = 0.731), and seven pairs exceeded 0.5 (including height–grip strength ρ = 0.689 and GDS-15–Kihon Checklist total ρ = 0.506). Multicollinearity was therefore not a material threat to model estimation, although correlated predictors remain a recognised source of instability in permutation-importance rankings (Section 3.7). Full results are in Supplementary Table S5.

### 3.3. Discrimination Across Model Families

**Figure 1:**
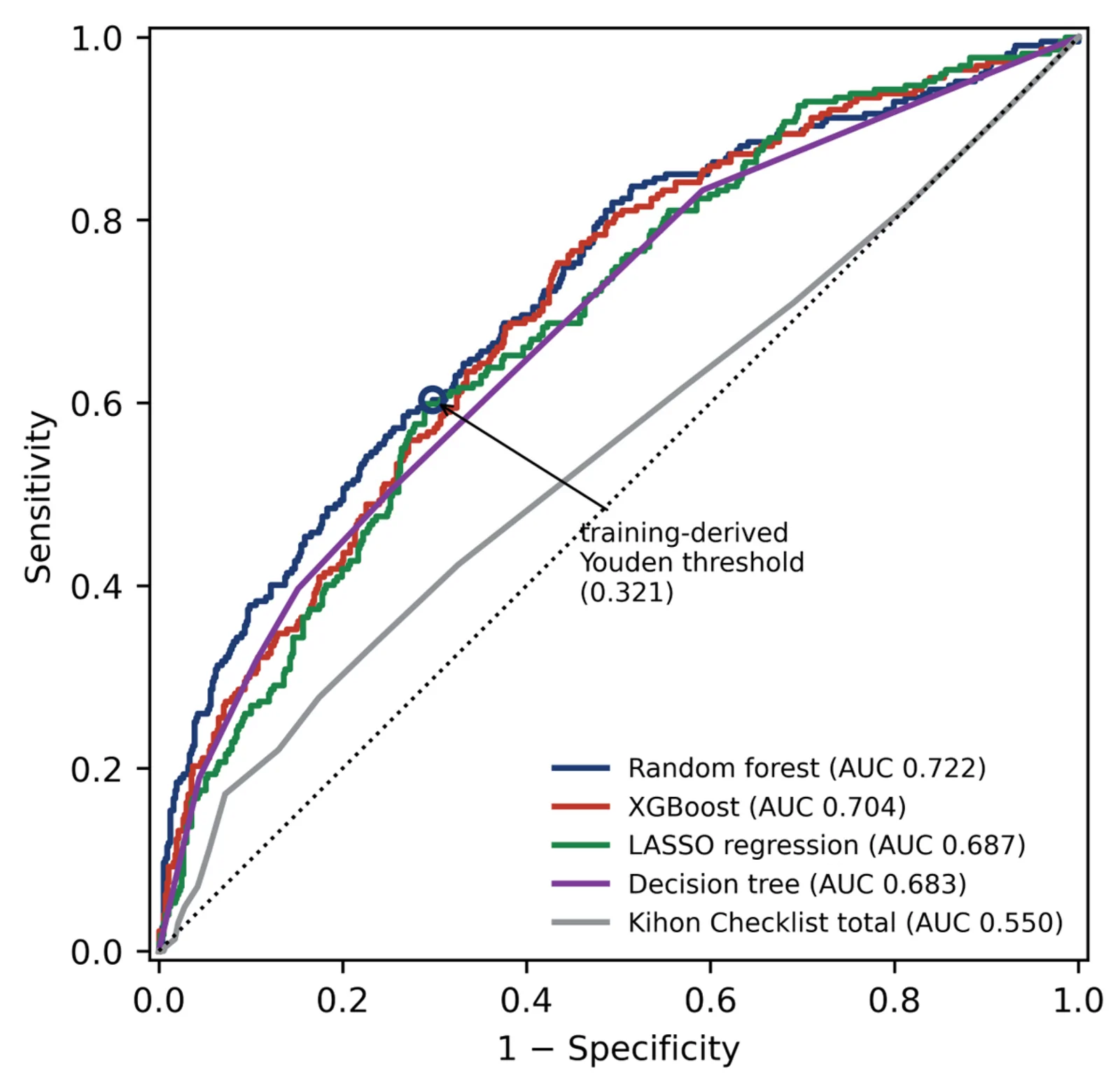
Receiver operating characteristic curves on the held-out test set (*n* = 795). The open circle marks the operating point corresponding to the Youden threshold derived from training out-of-fold predictions (0.321). The dotted diagonal line indicates the discrimination expected by chance (AUC 0.500).

**Figure 2:**
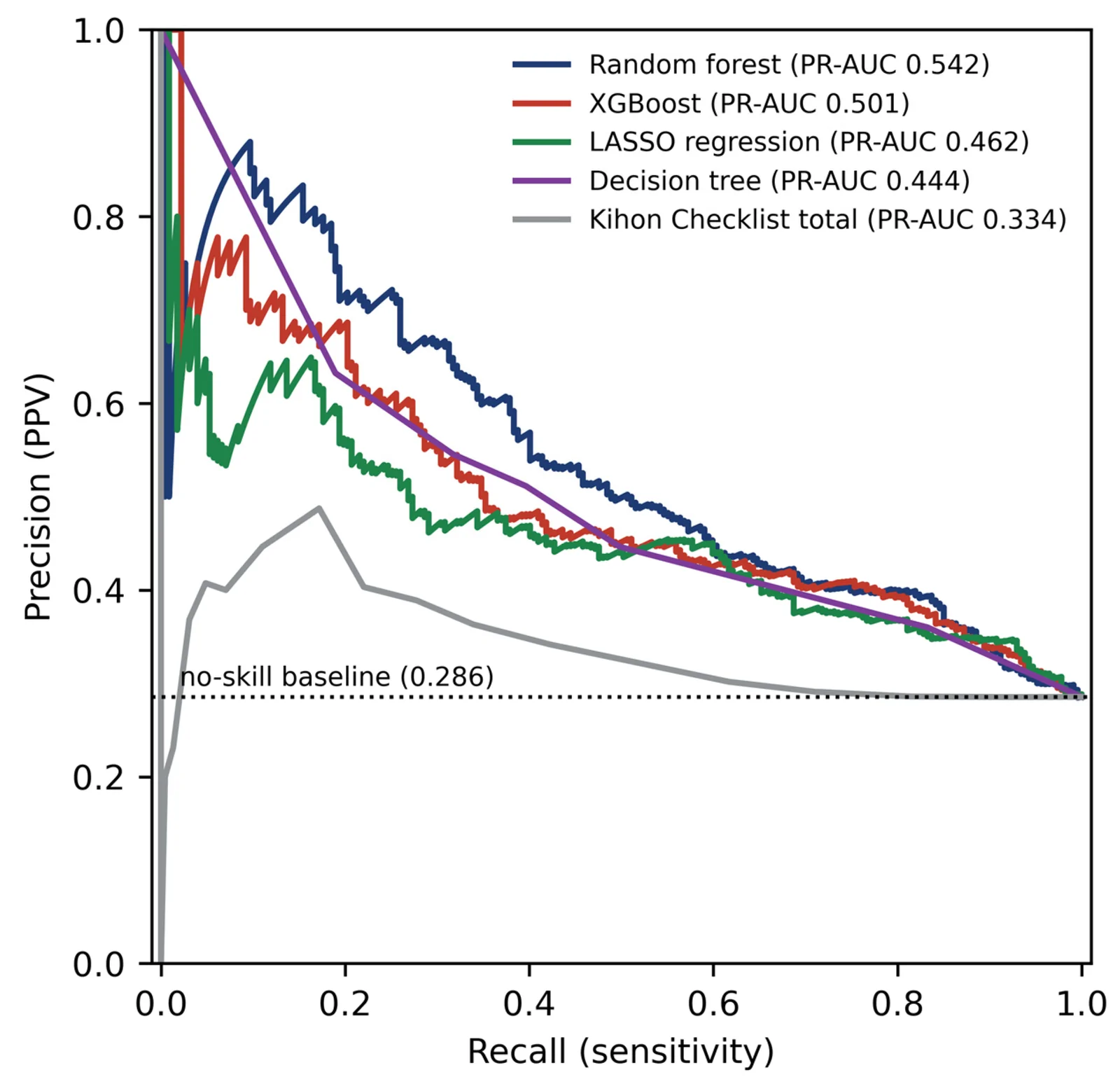
Precision–recall curves on the held-out test set. The dotted line is the no-skill baseline, equal to the MCI prevalence in the test set (0.286).

Test-set discrimination for all 16 models and reference scores is shown in Table 2. The random forest achieved the highest ROC AUC (0.722, 95% CI 0.681–0.761) and the highest PR-AUC (0.542), followed by the class-weighted random forest (0.718), cost-sensitive XGBoost (0.705) and XGBoost (0.704). Among regression-based models, LASSO logistic regression reached AUC 0.691 and elastic net 0.689. Non-tree ML models performed less well (support vector machine 0.677, k-nearest neighbours 0.682, naïve Bayes 0.663, neural network 0.643). The simplified seven-variable score achieved AUC 0.697, whereas single-item reference scores performed poorly (age alone 0.629; Kihon Checklist total 0.550). Receiver operating characteristic curves for the principal models are shown in Figure 1, and the corresponding precision–recall curves in Figure 2.

### 3.4. Statistical Comparison of Discrimination

Table 3 presents DeLong tests comparing the random forest with every other model. The random forest significantly outperformed unpenalised logistic regression (ΔAUC +0.047, *p* = 0.002), ridge regression (+0.040, *p* = 0.007), the support vector machine (+0.045, *p* = 0.003), k-nearest neighbours (+0.040, *p* = 0.014), the single decision tree (+0.039, *p* = 0.014), naïve Bayes (+0.059, *p* < 0.001), the neural network (+0.079, *p* < 0.001) and both single-item reference scores (*p* < 0.001).

Critically, the advantage over LASSO logistic regression was of borderline significance (+0.031, 95% CI +0.001 to +0.061, *p* = 0.044) and over elastic net similarly marginal (+0.032, *p* = 0.036), while differences versus XGBoost (+0.018, *p* = 0.059), the class-weighted random forest (+0.004, *p* = 0.485), cost-sensitive XGBoost (+0.017, *p* = 0.089) and the simplified seven-variable score (+0.024, *p* = 0.110) were not statistically significant. The evidence therefore supports the value of multivariable integration over single-item screening, but does not support a strong claim that tree-based nonlinear modelling is superior to well-specified penalised regression in these data.

### 3.5. Stability of Performance Across Resampling

Repeating the full pipeline across 100 independent stratified 70/30 splits (Table 4) gave mean AUCs of 0.703 (SD 0.016) for the class-weighted random forest, 0.701 (SD 0.017) for the random forest, 0.690 (SD 0.016) for XGBoost, 0.682 (SD 0.017) for LASSO regression and 0.650 (SD 0.018) for the single decision tree. The range across splits for the random forest was 0.663–0.746, so the value obtained on our primary split (0.722) lies in the upper part of the achievable range and should be regarded as a mildly optimistic point estimate; the resampled mean of approximately 0.70 is the more defensible summary.

**Figure 3:**
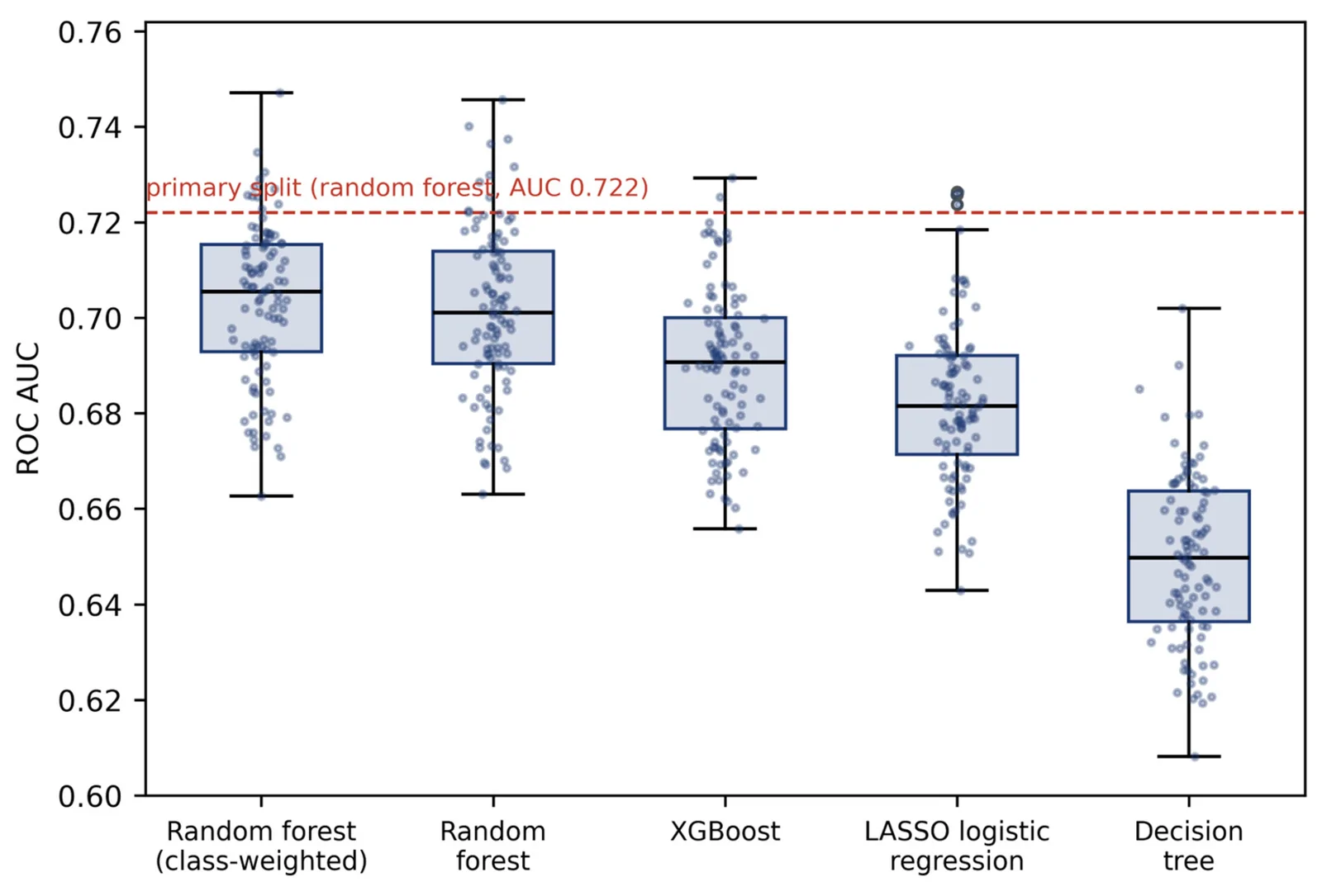
Distribution of test-set ROC AUC across 100 independent stratified 70/30 splits. Boxes show the median and interquartile range; points are individual splits. The dashed line marks the value obtained on the primary split reported in Table 2.

The ranking, however, was stable. Within-split paired differences showed the random forest exceeding LASSO regression in 94/100 splits (mean ΔAUC +0.020, SD 0.013) and XGBoost in 92/100 splits (mean +0.012, SD 0.009). Either random forest variant ranked first in 92/100 splits. Five-by-five repeated cross-validation on the full dataset reproduced the same ordering with much narrower variability (random forest 0.702, SD 0.006; class-weighted random forest 0.702, SD 0.005; XGBoost 0.689, SD 0.001; LASSO 0.679, SD 0.002; decision tree 0.651, SD 0.004). The distribution of AUC values across the 100 splits is shown in Figure 3.

### 3.6. Operating Points, Class Imbalance and Confusion Matrices

Table 5 reports test-set classification performance at three operating points, all determined from training data only.

At the default 0.5 threshold, the random forest correctly classified 61 of 227 MCI cases (sensitivity 0.269) while missing 166, with specificity 0.945 and accuracy 0.752. Accuracy at this threshold is therefore driven almost entirely by correct classification of the majority non-MCI class, and the default threshold is not usable for screening.

At the training-derived Youden threshold (0.321), sensitivity rose to 0.604 (137/227 cases detected), with specificity 0.699, positive predictive value 0.445, negative predictive value 0.815, F1 0.512 and balanced accuracy 0.651. In a test set of 795 attendees, this operating point would refer 308 individuals for cognitive assessment, of whom 137 (44.5%) would have MCI, while missing 90 cases.

At the training-derived 90%-sensitivity threshold (0.169), sensitivity reached 0.894 (203/227), but specificity fell to 0.301, referring 600 of 795 attendees—an unrealistic referral burden for most municipal programmes.

Class-weighted random forest behaved similarly (Youden threshold 0.330: sensitivity 0.612, specificity 0.692, PPV 0.443, F1 0.514), confirming that explicit class weighting improves the balance of errors modestly but does not overcome the fundamental discrimination ceiling.

Threshold transparency. In the initial submission of this manuscript, the reported Youden threshold (≈0.256, sensitivity 0.794, specificity 0.537) had been derived from the test-set ROC curve. In the present analysis, the test-derived Youden threshold for the random forest was 0.241, giving sensitivity 0.819 and specificity 0.507—closely reproducing the earlier figures and confirming that those figures were optimistic because the evaluation data had been used for threshold selection. All threshold-dependent results now reported are based on training-derived thresholds.

### 3.7. Calibration

**Figure 4:**
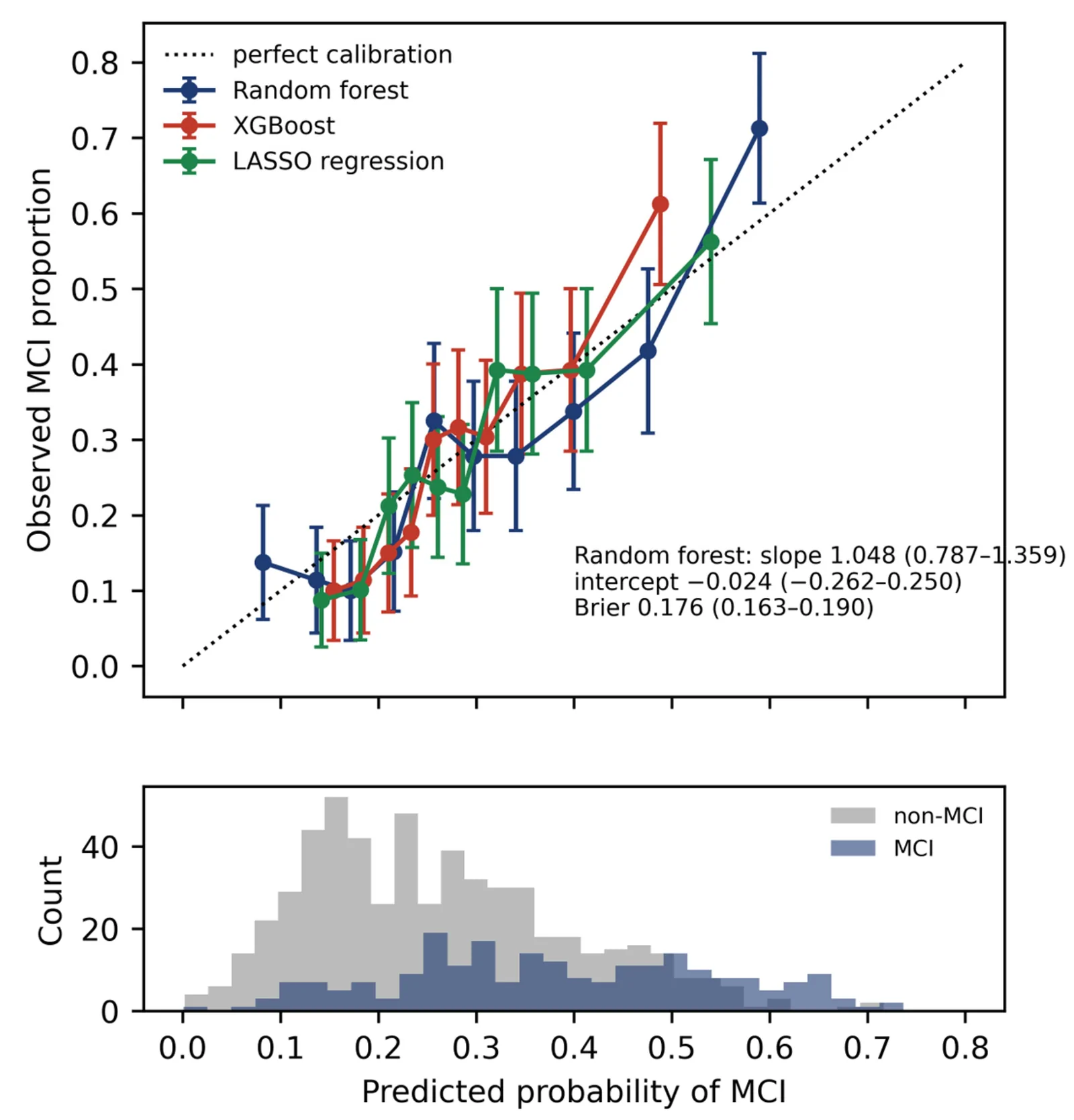
Calibration on the held-out test set. Upper panel: observed proportion of MCI against mean predicted probability by decile of predicted risk, with 95% confidence intervals; the dotted line indicates perfect calibration. Lower panel: distribution of random forest predicted probabilities by true MCI status.

Quantitative calibration metrics are reported in Table 6. The random forest was well calibrated: Brier score 0.176 (95% CI 0.163–0.190), calibration slope 1.048 (95% CI 0.787–1.359) and calibration intercept −0.024 (95% CI −0.262–0.250), with calibration-in-the-large −0.060 (95% CI −0.212–0.095). Both the slope confidence interval including 1 and the intercept interval including 0 indicate no evidence of systematic over- or under-fitting of predicted risks in this internal test set. XGBoost (slope 1.584) and LASSO regression (slope 1.584) showed calibration slopes significantly above 1, indicating under-dispersed predictions that would require recalibration before probability-based use. The calibration plot for the random forest is shown in Figure 4.

### 3.8. Clinical Utility: Decision-Curve Analysis

**Figure 5:**
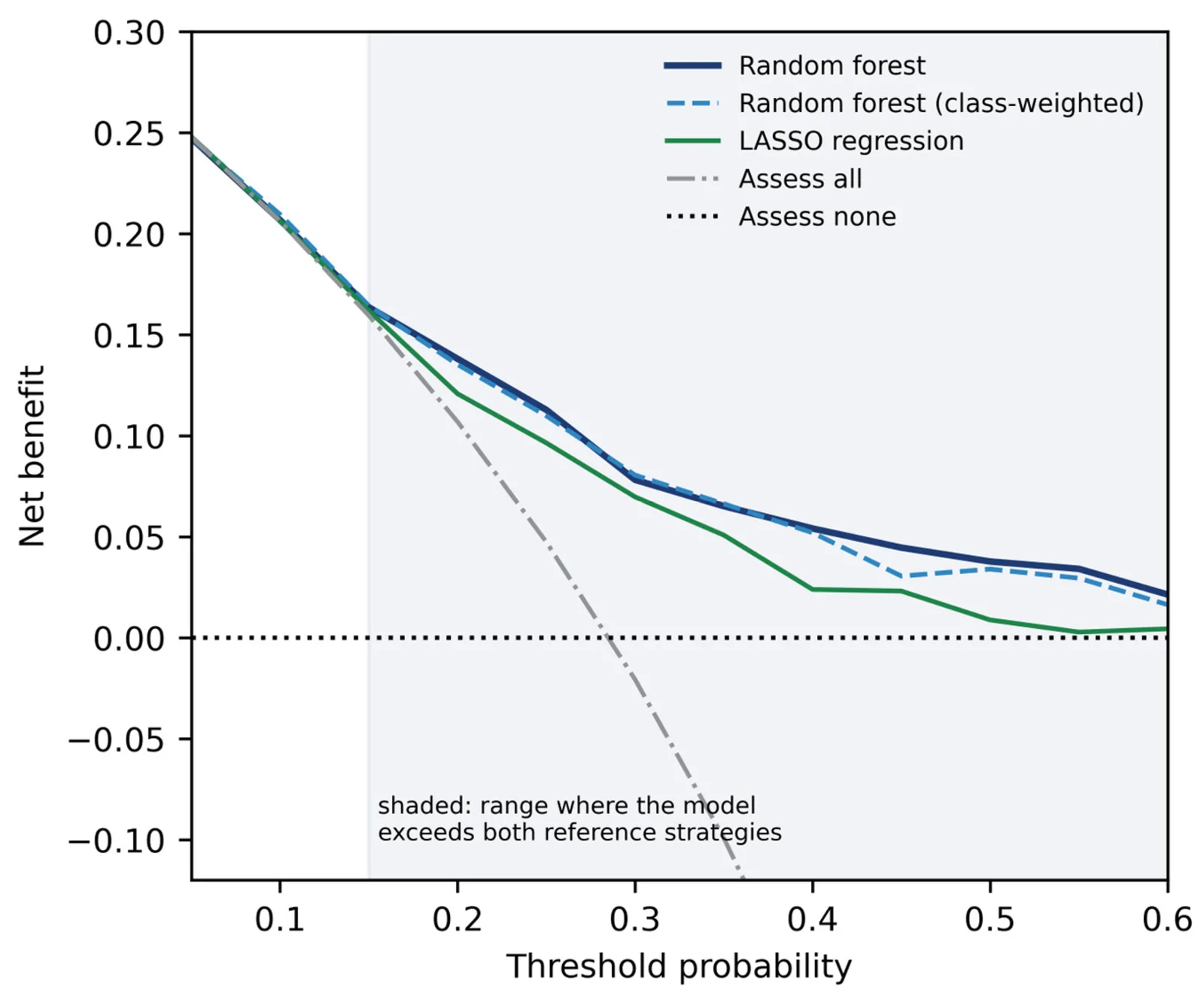
Decision-curve analysis on the held-out test set. Net benefit is plotted against threshold probability for the random forest, the class-weighted random forest and LASSO logistic regression, against the strategies of assessing all attendees and assessing none. The shaded band indicates the range in which the model exceeds both reference strategies.

Decision-curve analysis (Table 7, Figure 5) showed that the random forest yielded higher net benefit than both the assess-all and assess-none strategies across threshold probabilities from 0.15 to 0.60. The advantage over assess-all became substantial above the disease prevalence: at a threshold probability of 0.25, net benefit was 0.113 for the random forest versus 0.047 for assess-all, and at 0.30 it was 0.078 versus −0.021. Below a threshold probability of approximately 0.15—that is, when a missed MCI case is judged to be more than roughly six times as costly as an unnecessary cognitive assessment—assessing everyone was equivalent or preferable, which is an important and honest constraint on the model’s usefulness.

### 3.9. Variable Importance and Sensitivity Analyses

**Figure 6:**
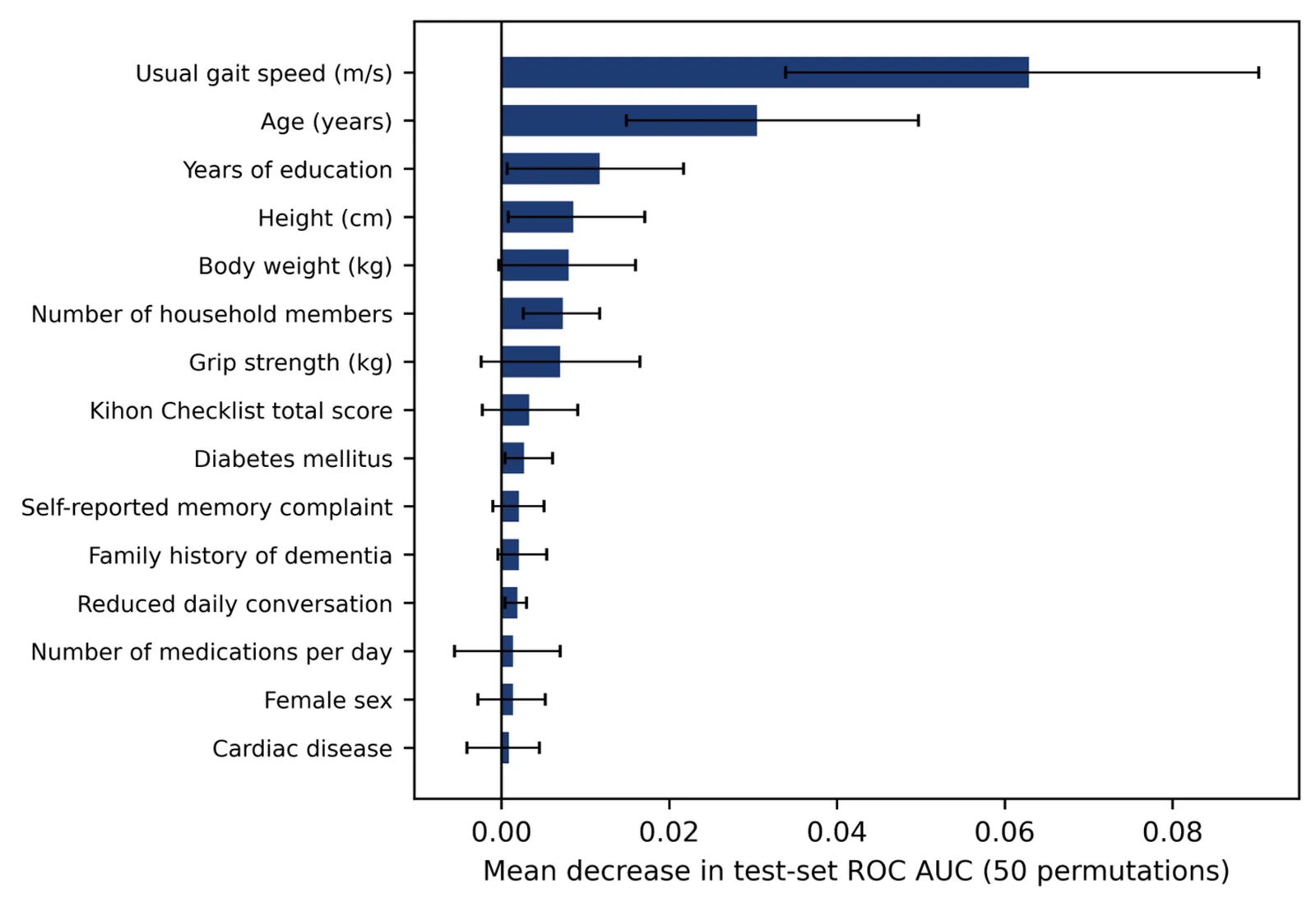
Permutation importance of the 15 highest-ranked predictors in the random forest, expressed as the mean decrease in test-set ROC AUC over 50 permutations, with 2.5th–97.5th percentile intervals. Bars in red denote predictors with non-positive mean importance.

Permutation importance for the random forest (Table 8, Figure 6) was dominated by usual gait speed (mean decrease in ROC AUC 0.063, 95% interval 0.034–0.090), followed by age (0.031, 0.015–0.050) and years of education (0.012, 0.001–0.022). Anthropometric and household variables contributed modestly, and individual comorbidities contributed little.

Nine of the 34 predictors had non-positive mean importance, and only seven had importance intervals excluding zero. Negative permutation importance does not mean that a variable is protective; it means that permuting the variable slightly improved test-set AUC, which occurs when a predictor carries essentially no signal and its inclusion adds noise. Consistent with the reviewers’ concern, we regard the importance ranking below approximately the seventh position as uninformative, and we explicitly recommend that variables with non-positive importance—including Parkinson’s disease, smoking status and several individual comorbidities—be dropped from future iterations of this model rather than being interpreted substantively.

Sensitivity analyses. Excluding all neurological and psychiatric diagnoses (Parkinson’s disease, physician-diagnosed depression, self-reported stroke) left discrimination essentially unchanged (AUC 0.724 vs. 0.722; PR-AUC 0.544 vs. 0.542), confirming that the model does not depend on conditions lying on the cognitive-decline spectrum. Excluding self-reported memory complaint gave AUC 0.720, and excluding both sets gave 0.720. A pre-specified parsimonious 12-predictor model retained most performance (AUC 0.702, PR-AUC 0.521; Supplementary Table S3) with a substantially reduced data-collection burden, and may be the more practical basis for future deployment.

Exploratory fuzzy inference system. The Wang–Mendel fuzzy rule base with three fuzzy sets per variable generated 135 interpretable rules over six linguistic variables and achieved test-set AUC 0.657 (PR-AUC 0.442); coarser partitions performed worse (four sets: 111 rules, AUC 0.625; five sets: 71 rules, AUC 0.614). Although discrimination was below that of the random forest, the rule base is directly readable by clinicians—for example, IF age is high AND education is low AND GDS-15 is high AND gait speed is low, THEN MCI risk = 0.84, versus IF age is medium AND education is high AND GDS-15 is low AND gait speed is medium AND physical frailty is low AND social frailty is low, THEN MCI risk = 0.00. The complete rule base is provided in Supplementary Table S4.

### 3.10. Risk of Bias and Applicability (PROBAST+AI)

The PROBAST+AI assessment is presented in full in Table 9. Overall risk of bias was judged high, driven principally by the analysis domain (single-region development sample, no external validation, and—in the initial submission—threshold optimisation on evaluation data, now corrected). Applicability was judged a low concern for the participants and predictors domains and a great concern for the outcome domain, because MCI was operationalised by a single computerised battery rather than by clinical consensus diagnosis.

## 4. Discussion

Using routinely collected community health check-up data from Akita Prefecture, we compared tree-based machine learning against regularised regression, other machine-learning families and simple reference scores for identifying MCI among community-dwelling older adults. Three findings should be emphasised, two of which qualify the conclusions of our initial submission.

### 4.1. Machine Learning Added Little over Regularised Regression

The random forest achieved the highest discrimination (AUC 0.722), but its advantage over LASSO logistic regression was +0.031 with a confidence interval reaching almost to zero (*p* = 0.044), and its advantage over gradient-boosted trees and over a simplified seven-variable score was not statistically significant. In 100 repeated splits, the random forest outperformed LASSO in 94/100 splits, so the ordering is reproducible; but the magnitude—approximately two AUC points—is small relative to what would be required to change clinical decisions.

We therefore now frame the contribution of this study as demonstrating the value of multidomain data integration rather than of algorithmic sophistication. The gap between any multivariable model (AUC 0.68–0.72) and the single-item screening currently used in Japanese municipalities (Kihon Checklist total score, AUC 0.550) is large and statistically unambiguous (ΔAUC 0.172, *p* < 0.001). This is the finding with practical implications: combining information already collected at a check-up substantially outperforms the single summary score currently in use, whether the combination is done by a random forest or by a penalised regression.

This conclusion is consistent with the broader prediction-modelling literature, in which flexible learners rarely outperform well-specified regression on low-dimensional tabular clinical data with modest sample sizes.

### 4.2. Comparison with Prior Studies

Our AUC of 0.722 (resampled mean 0.70) sits within the range reported for non-imaging, community-based MCI models. It is somewhat higher than the 0.673 reported for the best-performing model (also a random forest) in the Korean Longitudinal Study of Ageing, which used self-reported MCI diagnosis in 4975 participants [6], and lower than the 0.747 achieved in internal validation by a Chinese community nomogram that additionally incorporated serum creatinine and Traditional Chinese Medicine constitution typing at a similar MCI prevalence of 24.1% [7]. Models incorporating direct cognitive or imaging measures reach considerably higher discrimination [8], but by construction cannot be applied before cognitive testing has taken place.

Our influential predictors are also broadly concordant with prior work. Usual gait speed, age and education emerged as the strongest contributors, consistent with the extensive literature on motor–cognitive coupling and cognitive reserve, and with prior Japanese cohort work linking reduced mobility and social activity to cognitive impairment and frailty [21]. Psychosocial variables—household composition and reduced daily conversation—contributed measurably though modestly, echoing the KLoSA analysis in which living alone, being widowed and social engagement ranked among the strongest Shapley contributors for MCI [6]. Notably, the individual comorbidity variables that we had highlighted in our initial submission contributed little once continuous physical-function and demographic variables were properly included, and we have corrected the emphasis of the manuscript accordingly.

### 4.3. Threshold Selection Is the Central Practical Problem

The most important negative finding is that neither operating point is deployable as it stands. At the default threshold, 166 of 227 MCI cases (73%) were missed despite an accuracy of 0.752—an accuracy attributable almost entirely to the 71.4% non-MCI majority. At the training-derived Youden threshold, sensitivity of 0.604 came with a positive predictive value of 0.445, meaning that a little over half of those referred would not meet MCI criteria. Pushing sensitivity to 0.90 would require referring 75% of all attendees.

Concretely, for the Akita setting: a typical municipal check-up cohort of 800 attendees contains roughly 230 individuals with MCI. A formal NCGG-FAT assessment takes approximately 20 min of trained staff time. Operating at the Youden threshold would generate about 310 referrals (≈103 staff hours) and identify 137 cases while missing 90. Operating at the 90%-sensitivity threshold would generate about 600 referrals (≈200 staff hours) and identify 203 cases while missing 24. Given that most participating municipalities can currently resource on the order of 300–350 assessments per check-up cycle, the Youden operating point is the only one compatible with existing capacity, and the corresponding 40% miss rate must be regarded as the price of that constraint—a price that is acceptable only if the programme is repeated annually, so that cases missed in one cycle can be captured in the next. We would not regard a single-cycle miss rate of 40% as acceptable for a one-off screening programme. Municipalities with greater capacity should lower the threshold; those with less should not deploy the model at all, since below an approximately 0.15 threshold, decision-curve analysis shows no advantage over simply assessing everyone.

Decision-curve analysis nevertheless indicates that, within the 0.15–0.60 threshold range, model-guided triage delivers greater net benefit than either assessing all attendees or assessing none. This provides the first quantitative evidence of potential clinical utility for this model, while making its boundaries explicit.

### 4.4. Calibration and Interpretability

Unlike discrimination, calibration of the random forest was good (slope 1.048, intercept −0.024, Brier 0.176), with confidence intervals encompassing perfect calibration. This matters for the intended use: if the model is used to rank attendees for limited assessment slots, calibrated probabilities allow the referral threshold to be set rationally against capacity; calibration is increasingly recognised as the aspect of predictive performance most often neglected and most consequential for decision-making [22]. By contrast, XGBoost and LASSO regression showed calibration slopes significantly above 1, and would require recalibration before probability-based deployment.

The fuzzy inference system suggested by one reviewer produced a fully transparent, linguistically expressed rule base with only moderately reduced discrimination (AUC 0.657 vs. 0.722). We see genuine value in this direction. Fuzzy modelling offers a general platform in which the same underlying variables can be represented as graded linguistic categories rather than hard cut-points, which corresponds more closely to how clinicians reason about “somewhat slow gait” or “mildly low mood,” and which avoids the discontinuities introduced by dichotomised frailty and depression scores. It also provides a natural route to combining data-derived rules with expert-specified rules, and, as demonstrated in the fuzzy-systems literature on complex dynamical behaviour in coupled fuzzy models [23], to extending a static classifier into a dynamic model of trajectories over repeated check-up cycles. We regard the present rule base as a first step and plan to develop a general fuzzy modelling platform, incorporating longitudinal ORANGE Registry data, as a distinct line of work.

### 4.5. Prevalence and Selection

Our MCI prevalence of 28.6% is higher than the 24.1% reported in a comparable Chinese community sample [7] and higher than most population estimates for adults aged 65 and over. Two factors plausibly contribute. First, the NCGG-FAT with a 1.5 SD cut-off applied across multiple domains identifies a broader group than clinical consensus diagnosis, and multi-domain testing mechanically increases the probability that at least one domain falls below threshold. Second, and in the opposite direction, our exclusion criteria removed participants with dementia, Parkinson’s disease, other neurological diseases, major depression, stroke and certified long-term care needs, which should reduce observed prevalence. The net effect is that our sample is simultaneously healthier than the general older population in terms of comorbidity and more liberally classified in terms of cognition. Both features limit transportability: the model was developed on relatively healthy, independently mobile, voluntarily participating older adults, and its performance in a general community population that includes people with stroke, sensory impairment or care needs is unknown and could be either better (greater case heterogeneity) or worse (predictors behaving differently). This is a substantive limitation rather than a technicality.

### 4.6. Limitations

Beyond the issues already discussed, the following limitations apply. The design is cross-sectional, so the model classifies prevalent MCI rather than predicting incident MCI, and no causal interpretation of predictors is warranted. Data derive from a single prefecture, and no external validation was performed; the study therefore reports an internally validated exploratory model, not a validated screening framework, and this is reflected in the PROBAST+AI judgement of high overall risk of bias. Missing data were handled by single median imputation rather than multiple imputation, and several variables had missingness above 25%; multiple imputation with sensitivity analysis under alternative missingness assumptions is a priority for the next iteration. Outcome misclassification is possible given the operational MCI definition. Permutation importance is unstable in the presence of correlated predictors and should be read as a coarse ranking of the top few variables only. Finally, the recruitment strategy was voluntary, so participants are likely healthier and more health-conscious than non-participants. Numbers invited, attending and excluded were not recorded prospectively, so the degree of selection at the point of eligibility screening cannot be quantified.

### 4.7. Future Work

External validation is planned for FY2026–FY2027 using independent cohorts assembled outside Akita Prefecture from community-based health-check programmes and ORANGE Registry sites in other Japanese prefectures, where the same non-cognitive predictors are collected under comparable protocols. That work will (i) evaluate transportability of discrimination and calibration, (ii) refit using the parsimonious 12-predictor set with multiple imputation, (iii) recalibrate to local prevalence, and (iv) prospectively evaluate implementation outcomes—uptake of follow-up assessment, staff time, and unintended consequences such as anxiety among false-positive individuals—following DECIDE-AI [20].

## 5. Conclusions

In this cross-sectional study of 2650 community-dwelling older adults in Akita, Japan, models built from routinely collected non-cognitive health check-up data achieved moderate but well-calibrated discrimination for MCI, with the random forest reaching an ROC AUC of 0.722 on a held-out test set (resampled mean 0.70). The incremental advantage of tree-based machine learning over regularised logistic regression was small (ΔAUC +0.031) and of borderline statistical significance, so the substantive contribution lies in the integration of multiple routinely collected domains—which clearly outperformed the single-item screening currently in use—rather than in algorithmic complexity. Usual gait speed, age and education were the most influential predictors.

Neither the default nor the sensitivity-maximising operating point is clinically deployable: the former misses roughly three-quarters of cases and the latter would refer three-quarters of all attendees. At a training-derived Youden threshold, the model would refer 39% of attendees to detect 60% of cases at a positive predictive value of 0.445, which is compatible with current assessment capacity in participating Akita municipalities only within an annually repeated programme. Decision-curve analysis supports net benefit over assess-all and assess-none strategies between threshold probabilities of 0.15 and 0.60, but not below 0.15.

The model should therefore be understood as an internally validated exploratory triage aid. External validation in independent populations, recalibration to local prevalence, and prospective evaluation of implementation outcomes are prerequisites before any clinical or public-health deployment.

## Figures and Tables

**Table 1:** Characteristics of participants by MCI status.

Variable	Non-MCI (*n* = 1893)	MCI (*n* = 757)	*p* Value	SMD	Missing
Female sex, *n* (%)	1159 (68.9)	412 (64.3)	0.039	−0.097	326 (12.3%)
Age, years	75.4 (5.9)	78.0 (6.3)	<0.001	0.425	62 (2.3%)
Family history of dementia (0–2)	0.3 (0.5)	0.2 (0.4)	0.001	−0.161	189 (7.1%)
Hypertension, *n* (%)	878 (50.5)	399 (55.3)	0.034	0.096	192 (7.2%)
Stroke (self-reported), *n* (%)	15 (1.1)	19 (3.4)	0.001	0.152	763 (28.8%)
Cardiac disease, *n* (%)	370 (21.2)	163 (22.6)	0.497	0.032	186 (7.0%)
Diabetes mellitus, *n* (%)	271 (15.6)	141 (19.6)	0.018	0.105	190 (7.2%)
Hyperlipidaemia, *n* (%)	594 (34.2)	211 (29.3)	0.021	−0.105	190 (7.2%)
Osteoporosis, *n* (%)	407 (23.4)	168 (23.3)	1.000	−0.002	188 (7.1%)
Respiratory disease, *n* (%)	174 (10.0)	66 (9.2)	0.566	−0.029	190 (7.2%)
Knee osteoarthritis, *n* (%)	325 (18.7)	139 (19.3)	0.770	0.015	192 (7.2%)
Fracture after age 60, *n* (%)	188 (10.8)	81 (11.3)	0.793	0.015	191 (7.2%)
Malignant neoplasm, *n* (%)	294 (16.9)	111 (15.4)	0.410	−0.039	187 (7.1%)
Parkinson’s disease, *n* (%)	1 (0.1)	0 (0.0)	1.000	−0.039	802 (30.3%)
Alzheimer’s disease, *n* (%)	0 (0.0)	0 (0.0)	n.a.	0.000	816 (30.8%)
Physician-diagnosed depression, *n* (%)	4 (0.3)	3 (0.6)	0.735	0.036	836 (31.5%)
Rheumatoid arthritis/collagen disease, *n* (%)	86 (4.9)	29 (4.0)	0.378	−0.045	190 (7.2%)
Number of medications per day	3.3 (2.9)	3.7 (3.1)	0.003	0.138	244 (9.2%)
Years of education	12.2 (2.2)	11.5 (2.3)	<0.001	−0.326	185 (7.0%)
Number of household members	2.5 (1.4)	2.6 (1.5)	0.021	0.103	144 (5.4%)
Living alone, *n* (%)	411 (22.8)	151 (20.7)	0.285	−0.050	115 (4.3%)
Height, cm	154.9 (8.7)	154.3 (9.1)	0.132	−0.073	142 (5.4%)
Body weight, kg	57.1 (10.4)	57.3 (10.8)	0.301	0.019	144 (5.4%)
Usual gait speed, m/s	1.3 (0.3)	1.2 (0.3)	<0.001	−0.591	94 (3.5%)
Grip strength, kg	25.8 (7.8)	24.7 (8.1)	<0.001	−0.136	80 (3.0%)
≥5% weight loss in 2 years, *n* (%)	162 (9.3)	83 (11.7)	0.096	0.076	203 (7.7%)
Smoking status (1–3)	1.2 (0.5)	1.2 (0.5)	0.864	0.006	246 (9.3%)
GDS-15 score	2.9 (2.7)	3.2 (2.9)	0.001	0.136	185 (7.0%)
Kihon Checklist total score	4.1 (3.2)	4.7 (3.4)	<0.001	0.195	368 (13.9%)
Reduced daily conversation, *n* (%)	133 (7.4)	66 (9.1)	0.174	0.062	116 (4.4%)
Light exercise habit (1–5)	2.8 (1.3)	2.8 (1.4)	0.855	0.015	199 (7.5%)
Regular exercise habit (1–5)	3.6 (1.3)	3.7 (1.3)	0.292	0.034	195 (7.4%)
Self-reported memory complaint, *n* (%)	511 (30.1)	252 (36.7)	0.002	0.140	265 (10.0%)
Physical frailty status (0–2)	0.5 (0.6)	0.7 (0.6)	<0.001	0.336	372 (14.0%)
Social frailty status (0–2)	0.7 (0.8)	0.8 (0.8)	0.040	0.093	278 (10.5%)

Values are mean (SD) unless stated. *p* values from Mann–Whitney U tests (continuous) or chi-square tests (binary). SMD, standardised mean difference. Alzheimer’s disease had no recorded cases and was removed as a zero-variance predictor.

**Table 2:** Discrimination and overall accuracy of predicted probabilities on the held-out test set (*n* = 795).

Model	ROC AUC (95% CI)	PR-AUC	Brier Score
**Random forest**	**0.722 (0.681–0.761)**	**0.542**	**0.176**
Random forest (class-weighted)	0.718 (0.678–0.758)	0.518	0.178
XGBoost (cost-sensitive)	0.705 (0.664–0.745)	0.510	0.212
XGBoost	0.704 (0.664–0.744)	0.501	0.184
Simplified 7-variable score	0.697 (0.655–0.737)	0.490	n.a.
Logistic regression (LASSO, L1)	0.691 (0.649–0.730)	0.471	0.187
Logistic regression (elastic net)	0.689 (0.647–0.728)	0.469	0.188
Decision tree (CART)	0.683 (0.641–0.722)	0.444	0.184
k-nearest neighbours	0.682 (0.639–0.721)	0.450	0.192
Logistic regression (ridge, L2)	0.681 (0.639–0.722)	0.467	0.188
Support vector machine (RBF)	0.677 (0.635–0.720)	0.453	0.192
Logistic regression (unpenalised)	0.675 (0.632–0.716)	0.448	0.189
Naïve Bayes	0.663 (0.619–0.703)	0.425	0.713
Fuzzy inference system (3 sets/variable)	0.657 (n.a.)	0.442	n.a.
Neural network (MLP)	0.643 (0.600–0.684)	0.394	0.228
Age alone	0.629 (0.583–0.671)	0.420	n.a.
Kihon Checklist total score alone	0.550 (0.503–0.595)	0.334	n.a.

The 95% CIs from stratified bootstrap (2000 replicates). PR-AUC, area under the precision–recall curve; n.a., not applicable. The Brier score is not applicable to scores that are not expressed on a probability scale. Bold type indicates the model with the highest discrimination.

**Table 3:** DeLong comparisons of test-set ROC AUC: random forest versus each comparator. An em dash (—) indicates that the comparison is not applicable because the row is the reference model.

Comparator	AUC	ΔAUC (RF − Comparator)	95% CI	*p* Value
Random forest (class-weighted)	0.718	+0.004	−0.006 to +0.013	0.485
XGBoost (cost-sensitive)	0.705	+0.017	−0.003 to +0.036	0.089
XGBoost	0.704	+0.018	−0.001 to +0.037	0.059
Simplified 7-variable score	0.697	+0.024	−0.006 to +0.054	0.110
Logistic regression (LASSO)	0.691	+0.031	+0.001 to +0.061	0.044
Logistic regression (elastic net)	0.689	+0.032	+0.002 to +0.063	0.036
Decision tree (CART)	0.683	+0.039	+0.008 to +0.070	0.014
k-nearest neighbours	0.682	+0.040	+0.008 to +0.071	0.014
Logistic regression (ridge)	0.681	+0.040	+0.011 to +0.069	0.007
Support vector machine (RBF)	0.677	+0.045	+0.015 to +0.074	0.003
Logistic regression (unpenalised)	0.675	+0.047	+0.017 to +0.077	0.002
Naïve Bayes	0.663	+0.059	+0.026 to +0.092	<0.001
Neural network (MLP)	0.643	+0.079	+0.037 to +0.120	<0.001
Age alone	0.629	+0.093	+0.048 to +0.137	<0.001
Kihon Checklist total alone	0.550	+0.172	+0.118 to +0.225	<0.001

**Table 4:** Stability of ROC AUC across 100 repeated stratified 70/30 splits and 5 × 5 repeated cross-validation.

Model	Repeated Splits: Mean (SD)	Range	Splits Ranked 1st	Repeated 5 × 5 CV: Mean (SD)
Random forest (class-weighted)	0.703 (0.016)	0.663–0.747	57/100	0.702 (0.005)
Random forest	0.701 (0.017)	0.663–0.746	35/100	0.702 (0.006)
XGBoost	0.690 (0.016)	0.656–0.729	4/100	0.689 (0.001)
Logistic regression (LASSO)	0.682 (0.017)	0.643–0.726	3/100	0.679 (0.002)
Decision tree (CART)	0.650 (0.018)	0.608–0.702	1/100	0.651 (0.004)

**Table 5:** Test-set classification performance at operating points derived from training data only.

Model/Threshold	TP	FN	TN	FP	Accuracy	Sensitivity	Specificity	PPV	NPV	F1	Balanced Accuracy
RF, default 0.5	61	166	537	31	0.752	0.269	0.945	0.663	0.764	0.382	0.607
RF, training Youden (0.321)	137	90	397	171	0.672	0.604	0.699	0.445	0.815	0.512	0.651
RF, training sensitivity ≥ 0.90 (0.169)	203	24	171	397	0.470	0.894	0.301	0.338	0.877	0.491	0.598
RF class-weighted, default 0.5	59	168	536	32	0.748	0.260	0.944	0.648	0.761	0.371	0.602
RF class-weighted, training Youden (0.330)	139	88	393	175	0.669	0.612	0.692	0.443	0.817	0.514	0.652
RF class-weighted, training sensitivity ≥ 0.90 (0.183)	204	23	167	401	0.467	0.899	0.294	0.337	0.879	0.490	0.596
(For comparison) RF, test-derived Youden (0.241)	186	41	288	280	0.596	0.819	0.507	0.399	0.875	0.537	0.663

The final row is shown only to quantify the optimism of test-derived thresholding and is not proposed as a valid operating point. TP, true positive; FN, false negative; TN, true negative; FP, false positive; PPV, positive predictive value; NPV, negative predictive value; RF, random forest.

**Table 6:** Quantitative calibration on the held-out test set.

Model	Brier Score (95% CI)	Calibration Slope (95% CI)	Calibration Intercept (95% CI)	Calibration-in-the-Large (95% CI)
Random forest	0.176 (0.163–0.190)	1.048 (0.787–1.359)	−0.024 (−0.262–0.250)	−0.060 (−0.212–0.095)
Random forest (class-weighted)	0.178 (0.166–0.192)	1.104 (0.837–1.363)	−0.030 (−0.256–0.231)	−0.103 (−0.264–0.068)
Decision tree (CART)	0.184 (0.170–0.200)	0.978 (0.751–1.215)	−0.012 (−0.271–0.250)	0.007 (−0.165–0.158)
XGBoost	0.184 (0.171–0.197)	1.584 (1.250–1.947)	0.495 (0.161–0.826)	−0.003 (−0.166–0.152)
Logistic regression (LASSO)	0.187 (0.176–0.199)	1.584 (1.233–1.987)	0.332 (0.028–0.687)	−0.110 (−0.261–0.033)

**Table 7:** Net benefit by threshold probability (test set).

Threshold Probability	Assess All	Assess None	Random Forest	RF (Class-Weighted)	LASSO Regression	Simplified 7-Variable Score
0.10	0.206	0	0.207	0.210	0.206	0.207
0.15	0.160	0	0.164	0.164	0.162	0.170
0.20	0.107	0	0.138	0.135	0.121	0.130
0.25	0.047	0	0.113	0.110	0.096	0.096
0.30	−0.021	0	0.078	0.081	0.070	0.072
0.35	−0.099	0	0.065	0.066	0.051	0.049
0.40	−0.191	0	0.054	0.052	0.024	0.031
0.50	−0.429	0	0.038	0.034	0.009	0.009
0.60	−0.786	0	0.021	0.016	0.004	0.000

**Table 8:** Permutation importance (mean decrease in test-set ROC AUC, 50 repetitions).

Rank	Variable	Importance	95% Interval
1	Usual gait speed	0.0629	0.0339 to 0.0903
2	Age	0.0305	0.0149 to 0.0497
3	Years of education	0.0117	0.0007 to 0.0217
4	Height	0.0086	0.0008 to 0.0171
5	Body weight	0.0080	−0.0003 to 0.0160
6	Number of household members	0.0073	0.0026 to 0.0117
7	Grip strength	0.0070	−0.0024 to 0.0165
8	Kihon Checklist total score	0.0033	−0.0023 to 0.0091
9	Diabetes mellitus	0.0027	0.0004 to 0.0061
10	Self-reported memory complaint	0.0021	−0.0010 to 0.0051
11	Family history of dementia	0.0021	−0.0004 to 0.0054
12	Reduced daily conversation	0.0019	0.0004 to 0.0030
13	Number of medications per day	0.0014	−0.0056 to 0.0070
14	Female sex	0.0014	−0.0028 to 0.0052
15	GDS-15 score	0.0007	−0.0050 to 0.0061

**Table 9:** PROBAST+AI assessment.

Domain	Signalling Issues Considered	Risk of Bias	Applicability
1. Participants	Voluntary community check-up attendees rather than a probability sample; exclusion criteria remove less healthy older adults, including all participants with dementia, Parkinson’s disease, other neurological disease, stroke or certified long-term care need; data pooled across municipalities and years (2018–2025)	High	Low concern
2. Predictors	Predictors pre-specified, routinely collected, measured identically across sites, assessed at the same visit as the outcome and without knowledge of cognitive test results; no data-driven pre-screening; VIF <3 for all predictors	Low	Low concern
3. Outcome	MCI defined by NCGG-FAT with age/education-adjusted norms and a 1.5 SD cut-off, applied uniformly; no clinical consensus diagnosis or biomarker confirmation; outcome assessors not blinded to predictor data	High	High concern
4. Analysis	Adequate events per predictor (757 events, 34 predictors); missing data handled by training-set median imputation without multiple imputation; complete separation of training and test data including for threshold derivation (corrected in this revision); discrimination, calibration and net benefit all reported; stability assessed by 100 repeated splits and 5 × 5 repeated CV; no external validation	High	High concern
**Overall**	—	**High**	**High concern**

## Data Availability

The analysis code is available from the corresponding author on reasonable request. Individual participant data cannot be shared publicly owing to the terms of participant consent and municipal data-sharing agreements.

## Supplementary Materials

The following supporting information can be downloaded at: https://www.mdpi.com/article/10.3390/geriatrics11050138/s1, Figure S1: participant flow diagram; Table S1: Final tuned hyperparameters, preprocessing specification and software versions for all models compared; Table S2: Complete list of the 34 candidate predictors with definitions, measurement procedures, coding, plausible ranges used for data-quality screening, and missingness.; Table S3: Sensitivity analyses of the random forest model on the primary training/test split, using alternative predictor sets; Table S4: Complete fuzzy rule base of the exploratory Mamdani-type inference system (135 rules over six linguistic variables); Table S5: Variance inflation factors for all candidate predictors, with a summary of the pairwise Spearman correlations.

## Abbreviations

AUC: area under the curve; CART, classification and regression tree; CI, confidence interval; DECIDE-AI, Developmental and Exploratory Clinical Investigations of Decision support systems driven by Artificial Intelligence; FN, false negative; FP, false positive; GDS-15, 15-item Geriatric Depression Scale; MCI, mild cognitive impairment; ML, machine learning; MLP, multilayer perceptron; NCGG-FAT, National Center for Geriatrics and Gerontology Functional Assessment Tool; NPV, negative predictive value; PPV, positive predictive value; PR-AUC, area under the precision–recall curve; PROBAST+AI, Prediction model Risk Of Bias Assessment Tool–AI extension; RF, random forest; ROC, receiver operating characteristic; SMD, standardised mean difference; TN, true negative; TP, true positive; TRIPOD+AI, Transparent Reporting of a multivariable prediction model for Individual Prognosis Or Diagnosis–AI extension; VIF, variance inflation factor.
