# Supplementary material for: Identifying Mild Cognitive Impairment from Routinely Collected Physical, Functional and Psychosocial Measures in Community-Dwelling Older Adults: A Comparative Machine-Learning and Regression Modelling Study in the Northern Japanese ORANGE Registry
Source: Geriatrics (Basel). 2026 Oct 1;11(5):138. doi: 10.3390/geriatrics11050138 (PMC13704611; doi:10.3390/geriatrics11050138)
Supplement: Supplementary file 1 [file geriatrics-11-00138-s001.zip › geriatrics-4501632-supplementary.pdf]

Supplementary Materials

Figure S1. Participant flow diagram

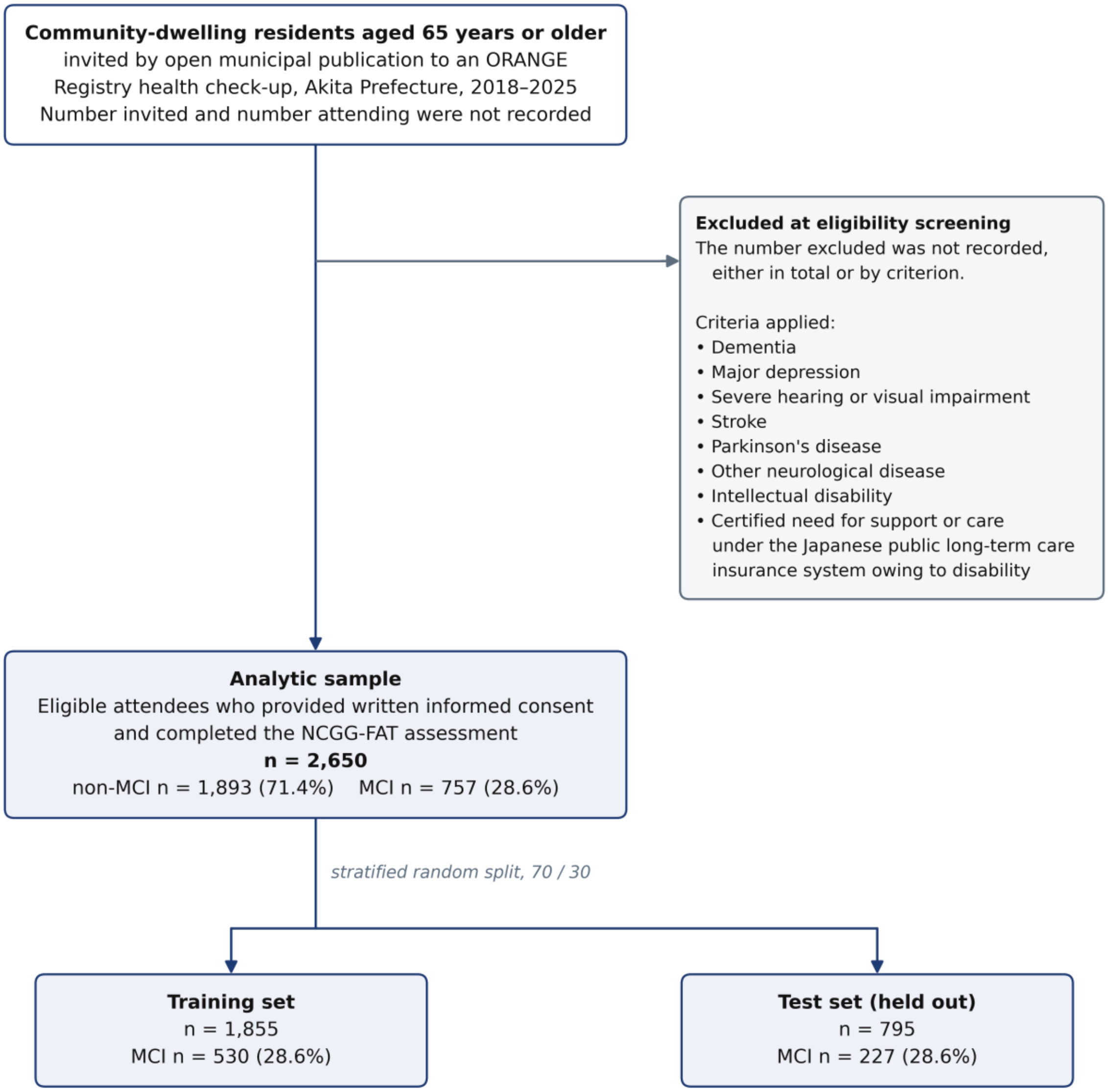

Figure S1. Flow of participants through the ORANGE Registry community health check-up programme, 2018–2025. Exclusion criteria were applied at eligibility screening.

Table S1. Final tuned hyperparameters, preprocessing specification and software versions for all models compared.

Model	Engine	Hyperparameter	Final value
Decision tree (CART)	decision_tree / rpart	cost_complexity	1 x 10^{-10}
		tree_depth	3
		min_n	5
Random forest	rand_forest / ranger	mtry	7
		trees	906
		min.node.size	4
		importance	permutation
XGBoost	boost_tree / xgboost	mtry	11
		trees	338
		min_n	3
		tree_depth	6
		learn_rate	0.00663
		loss_reduction	4.01 x 10^{-6}
		sample_size	0.598
Random forest (class-weighted)	rand_forest / ranger	class.weights	balanced sub-sampling
XGBoost (cost-sensitive)	boost_tree / xgboost	scale_pos_weight	2.50
Logistic regression (ridge)	logistic_reg / glmnet	penalty (mixture = 0)	tuned, 5-fold CV
Logistic regression (LASSO)	logistic_reg / glmnet	penalty (mixture = 1)	tuned, 5-fold CV
Logistic regression (elastic net)	logistic_reg / glmnet	penalty, mixture	tuned, 5-fold CV
Support vector machine (RBF)	svm_rbf / kernlab	cost, rbf_sigma	tuned, 5-fold CV
k-nearest neighbours	nearest_neighbor / kknn	neighbors	tuned, 5-fold CV
Naive Bayes	naive_Bayes / klaR	smoothness, Laplace	default
Neural network (MLP)	mlp / nnet	hidden_units, penalty	tuned, 5-fold CV
Fuzzy inference system	custom (Wang-Mendel)	fuzzy sets per variable	3 (4 and 5 also evaluated)
		minimum rule support	5 records

Table S1 footnote. Preprocessing (identical for every model, estimated on the training set only): screening of implausible values against the ranges in Table S2 and recoding as missing; median imputation of missing numeric values; removal of zero-variance predictors; z-score standardisation. Hyperparameter tuning used stratified five-fold cross-validation within the training set with ROC AUC as the selection criterion.

Software. Analyses were performed in R version 4.5.2 (R Foundation for Statistical Computing, Vienna, Austria) using the tidymodels framework, with the engines ranger version 0.17.0, rpart version 4.1.24, xgboost version 3.1.2.1, glmnet version 4.1-10, kernlab, kknn and nnet, and the packages pROC and dcurves. Results were cross-checked in Python version 3.12.3 (Python Software Foundation, Wilmington, DE, USA) using scikit-learn version 1.8.0 and xgboost version 3.4.1.

Table S2. Complete list of the 34 candidate predictors with definitions, measurement procedures, coding, plausible ranges used for data-quality screening, and missingness.

Variable	Definition / measurement	Coding	Plausible range	Missing, n (%)	Note
Female sex	Self-reported sex	1 = female, 0 = male	1-2	326 (12.3%)	
Age (years)	Age at assessment, from date of birth	years	60-110	62 (2.3%)	
Family history of dementia	Self-reported family history of dementia	0 = none, 1-2 = number of affected relatives	0-2	189 (7.1%)	
Hypertension	Self-reported physician diagnosis	0 = no, 1 = yes	0-1	192 (7.2%)	
Stroke (self-reported)	Self-reported physician diagnosis	0 = no, 1 = yes	0-1	763 (28.8%)	
Cardiac disease	Self-reported physician diagnosis	0 = no, 1 = yes	0-1	186 (7.0%)	
Diabetes mellitus	Self-reported physician diagnosis	0 = no, 1 = yes	0-1	190 (7.2%)	
Hyperlipidaemia	Self-reported physician diagnosis	0 = no, 1 = yes	0-1	190 (7.2%)	
Osteoporosis	Self-reported physician diagnosis	0 = no, 1 = yes	0-1	188 (7.1%)	
Respiratory disease	Self-reported physician diagnosis	0 = no, 1 = yes	0-1	190 (7.2%)	
Knee osteoarthritis	Self-reported physician diagnosis	0 = no, 1 = yes	0-1	192 (7.2%)	
Fracture after age 60	Self-reported fracture at or after age 60	0 = no, 1 = yes	0-1	191 (7.2%)	
Malignant neoplasm	Self-reported history of malignancy	0 = no, 1 = yes	0-1	187 (7.1%)	
Parkinson's disease	Self-reported physician diagnosis	0 = no, 1 = yes	0-1	802 (30.3%)	
Alzheimer's disease	Self-reported physician diagnosis	0 = no, 1 = yes	0-1	816 (30.8%)	excluded (zero variance: no recorded cases)
Physician-diagnosed depression	Self-reported physician diagnosis	0 = no, 1 = yes	0-1	836 (31.5%)	
Rheumatoid arthritis / collagen disease	Self-reported physician diagnosis	0 = no, 1 = yes	0-1	190 (7.2%)	

Variable	Definition / measurement	Coding	Plausible range	Missing, n (%)	Note
Number of medications per day	Number of different medications taken daily, from medication record	count	0-30	244 (9.2%)	
Years of education	Total years of formal schooling	years	0-25	185 (7.0%)	
Number of household members	Number of people in the household including the participant	count	0-12	144 (5.4%)	
Living alone	Living alone	0 = no, 1 = yes	0-1	115 (4.3%)	
Height (cm)	Measured standing height	cm	120-200	142 (5.4%)	
Body weight (kg)	Measured body weight	kg	25-150	144 (5.4%)	
Usual gait speed (m/s)	Usual gait speed over a marked course, single trial	m/s	0.1-3.0	94 (3.5%)	
Grip strength (kg)	Maximum handgrip strength, dominant hand, measured with a Smedley-type handgrip dynamometer (Takei Scientific Instruments Co., Ltd., Kamo city, Japan)	kg	0-80	80 (3.0%)	
>=5% weight loss in 2 years	Self-reported unintentional weight loss of 5% or more over 2 years	0 = no, 1 = yes	0-1	203 (7.7%)	
Smoking status	Smoking status	1 = never, 2 = former, 3 = current	1-3	246 (9.3%)	
GDS-15 score	15-item Geriatric Depression Scale	0-15, higher = more depressive symptoms	0-15	185 (7.0%)	
Kihon Checklist total score	Kihon Checklist total score	0-25, higher = greater functional decline	0-25	368 (13.9%)	
Reduced daily conversation	Reduced daily conversation (Makizako Social Frailty Index item)	0 = no, 1 = yes	0-1	116 (4.4%)	
Light exercise habit (frequency)	Frequency of light physical activity	1-5 ordinal, higher = more frequent	1-5	199 (7.5%)	
Regular exercise habit (frequency)	Frequency of regular structured exercise	1-5 ordinal, higher = more frequent	1-5	195 (7.4%)	
Self-reported	Self-reported memory complaint	1 = complaint	1-2	265	

Variable	Definition / measurement	Coding	Plausible range	Missing, n (%)	Note
memory complaint	(single item)	present, 0 = absent		(10.0%)	
Physical frailty status	Physical frailty, Fried phenotype (NCGG-SGS operationalisation)	0 = robust, 1 = pre-frail, 2 = frail	0-2	372 (14.0%)	
Social frailty status	Social frailty, Makizako Social Frailty Index	0 = robust, 1 = pre-frail, 2 = frail	0-2	278 (10.5%)	

Table S2 footnote. Values falling outside the plausible range were recoded as missing before imputation; this affected 139 of 90,100 cells (0.15%). Alzheimer's disease had no recorded cases in the assembled dataset and was removed as a zero-variance predictor, leaving 34 predictors.

Table S3. Sensitivity analyses of the random forest model on the primary training/test split, using alternative predictor sets.

Predictor set	Predictors, n	ROC AUC	PR-AUC
Full pre-specified set (primary model)	34	0.722	0.542
Excluding neurological / psychiatric diagnoses (Parkinson's disease, depression, stroke)	31	0.724	0.544
Excluding self-reported memory complaint	33	0.720	0.538
Excluding both of the above	30	0.720	0.535
Pre-specified parsimonious set	12	0.702	0.521

Table S3 footnote. The parsimonious set comprised age, sex, years of education, GDS-15 score, usual gait speed, grip strength, physical frailty status, social frailty status, living alone, osteoporosis, Kihon Checklist total score and number of medications per day.

Table S4. Complete fuzzy rule base of the exploratory Mamdani-type inference system (135 rules over six linguistic variables).

Mamdani-type inference system generated from the training set by the Wang-Mendel procedure, with three Gaussian membership functions (low / medium / high) per linguistic variable and a minimum rule support of five records. 135 rules were retained. Antecedents are listed in the order: Age / Education / GDS-15 / Gait speed / Physical frailty / Social frailty. The consequent is the membership-weighted mean MCI proportion among training records matching the antecedent. Rules are ordered by descending consequent.

Rule	Antecedent (Age / Education / GDS-15 / Gait speed / Physical frailty / Social frailty)	Consequent (MCI risk)	Rule weight	Support, n
1	high / low / high / low / medium / high	0.965	0.0	7
2	medium / high / high / medium / medium / high	0.954	0.3	5
3	high / medium / medium / high / medium / high	0.850	1.3	5
4	high / low / high / low / medium / low	0.836	2.6	8

Rule	Antecedent (Age / Education / GDS-15 / Gait speed / Physical frailty / Social frailty)	Consequent (MCI risk)	Rule weight	Support, n
5	high / medium / low / medium / medium / low	0.775	7.2	11
6	high / medium / high / low / medium / medium	0.770	1.9	8
7	medium / medium / high / low / medium / high	0.738	3.2	9
8	high / medium / low / low / medium / low	0.727	2.7	6
9	medium / low / high / medium / medium / medium	0.724	1.8	6
10	high / low / medium / low / medium / high	0.690	0.2	8
11	medium / medium / medium / low / low / medium	0.679	2.3	5
12	high / low / low / low / medium / low	0.649	4.4	9
13	high / medium / medium / low / medium / high	0.620	1.2	6
14	high / medium / low / low / low / low	0.606	2.8	5
15	high / low / medium / low / medium / low	0.602	4.5	11
16	high / medium / high / low / medium / low	0.543	3.0	9
17	high / low / high / medium / medium / medium	0.539	3.0	9
18	medium / medium / medium / low / medium / medium	0.538	7.7	16
19	medium / medium / medium / low / medium / high	0.530	1.5	6
20	low / high / medium / medium / medium / low	0.511	3.2	6
21	high / low / low / low / medium / medium	0.501	1.7	6
22	high / medium / medium / medium / medium / high	0.493	1.7	5
23	medium / low / medium / medium / low / low	0.487	6.8	11
24	low / low / medium / medium / low / low	0.484	4.7	8
25	high / medium / medium / medium / low / medium	0.480	6.9	11
26	medium / low / high / low / medium / high	0.476	0.8	7
27	low / high / medium / medium / low / low	0.472	2.1	6
28	medium / low / low / medium / low / low	0.469	7.1	11
29	high / medium / medium / low / medium / medium	0.462	7.0	19
30	high / low / medium / low / medium / medium	0.446	10.0	25
31	medium / medium / high / low / medium / medium	0.445	2.5	8
32	medium / medium / medium / low / low / low	0.439	7.6	14
33	medium / medium / low / medium / low / medium	0.437	10.2	14
34	low / medium / high / medium / medium / medium	0.428	4.9	10
35	high / high / low / medium / low / medium	0.423	3.2	5
36	high / low / high / low / medium / medium	0.422	3.4	16
37	high / medium / low / low / medium / medium	0.422	4.2	12
38	high / medium / medium / medium / medium / medium	0.416	7.5	12
39	medium / high / medium / low / medium / medium	0.413	2.8	7
40	high / medium / high / medium / medium / high	0.412	2.4	5
41	low / low / low / medium / low / low	0.411	4.5	6
42	high / low / medium / medium / medium / medium	0.408	3.4	5

Rule	Antecedent (Age / Education / GDS-15 / Gait speed / Physical frailty / Social frailty)	Consequent (MCI risk)	Rule weight	Support, n
43	medium / low / medium / low / medium / medium	0.404	3.7	11
44	medium / high / low / medium / low / medium	0.404	3.0	5
45	high / medium / high / low / medium / high	0.403	1.5	11
46	low / high / low / high / medium / low	0.390	3.1	5
47	medium / low / low / medium / medium / medium	0.387	8.1	11
48	medium / medium / medium / low / medium / low	0.387	11.9	24
49	high / medium / low / medium / medium / medium	0.368	8.3	13
50	medium / medium / medium / medium / medium / high	0.358	7.6	13
51	medium / medium / medium / medium / low / low	0.355	15.9	25
52	high / medium / medium / medium / medium / low	0.354	4.3	7
53	high / medium / medium / medium / low / low	0.347	3.9	7
54	low / low / medium / high / medium / medium	0.342	3.1	5
55	high / medium / medium / low / medium / low	0.336	5.9	12
56	low / medium / medium / medium / low / low	0.325	8.4	14
57	medium / medium / medium / medium / medium / medium	0.322	24.3	35
58	low / medium / high / medium / medium / high	0.315	2.6	9
59	low / medium / high / medium / low / medium	0.315	3.6	6
60	low / high / low / high / low / low	0.302	7.1	18
61	medium / low / high / medium / medium / high	0.297	2.6	5
62	high / medium / low / medium / low / medium	0.284	7.8	11
63	high / low / low / medium / low / low	0.284	5.1	7
64	medium / medium / high / medium / low / medium	0.280	4.7	9
65	medium / medium / low / medium / medium / low	0.280	10.1	16
66	medium / low / low / medium / medium / low	0.280	3.3	6
67	medium / medium / medium / high / low / low	0.274	7.2	14
68	medium / high / medium / medium / low / medium	0.265	3.1	6
69	low / low / medium / medium / medium / medium	0.262	2.5	5
70	medium / medium / medium / high / low / high	0.260	2.6	5
71	low / high / low / high / low / medium	0.244	2.7	5
72	medium / medium / high / medium / medium / high	0.243	5.9	12
73	medium / low / high / low / medium / medium	0.242	1.6	6
74	medium / low / medium / medium / medium / medium	0.231	4.5	7
75	medium / high / medium / medium / medium / medium	0.227	5.3	10
76	low / medium / medium / medium / medium / low	0.226	10.8	18
77	low / medium / medium / medium / low / medium	0.219	10.1	15
78	medium / medium / medium / medium / low / high	0.213	4.8	8
79	high / high / low / medium / medium / low	0.212	2.8	5
80	low / medium / low / medium / low / low	0.212	17.1	27

Rule	Antecedent (Age / Education / GDS-15 / Gait speed / Physical frailty / Social frailty)	Consequent (MCI risk)	Rule weight	Support, n
81	medium / low / low / high / low / low	0.204	3.9	6
82	medium / medium / low / low / medium / low	0.193	3.4	6
83	medium / low / medium / low / medium / low	0.193	3.0	9
84	low / medium / medium / medium / medium / medium	0.193	12.4	21
85	low / medium / low / high / low / medium	0.189	3.4	7
86	medium / medium / low / medium / low / low	0.185	18.4	29
87	low / medium / high / high / low / low	0.182	1.8	5
88	low / medium / low / medium / medium / low	0.182	5.4	7
89	medium / low / medium / medium / medium / low	0.167	3.8	6
90	medium / medium / medium / medium / low / medium	0.164	10.7	18
91	medium / medium / medium / high / medium / medium	0.159	17.5	25
92	low / high / low / medium / low / low	0.156	4.2	7
93	medium / high / medium / medium / medium / low	0.155	2.5	5
94	medium / medium / medium / high / medium / low	0.154	9.1	19
95	low / medium / low / medium / low / medium	0.154	7.3	12
96	medium / medium / low / medium / medium / medium	0.153	15.3	22
97	medium / high / medium / high / medium / medium	0.147	3.6	6
98	low / medium / low / high / low / low	0.142	12.3	20
99	medium / medium / high / medium / medium / low	0.141	3.5	8
100	low / medium / low / low / low / low	0.140	3.4	6
101	medium / medium / low / high / low / low	0.135	15.0	24
102	low / medium / medium / medium / medium / high	0.128	3.0	6
103	high / low / low / medium / medium / low	0.128	3.4	6
104	low / high / medium / high / medium / medium	0.122	3.2	9
105	medium / medium / low / low / medium / medium	0.114	4.3	9
106	high / medium / high / medium / medium / medium	0.110	2.2	6
107	low / medium / low / medium / medium / medium	0.106	5.7	9
108	medium / medium / high / medium / medium / medium	0.102	4.1	9
109	low / medium / medium / high / medium / medium	0.101	12.1	25
110	medium / medium / medium / high / low / medium	0.088	5.5	8
111	medium / medium / low / high / low / medium	0.080	9.5	14
112	low / medium / medium / high / medium / low	0.075	6.4	14
113	high / medium / low / medium / low / low	0.059	13.4	19
114	medium / medium / medium / medium / medium / low	0.057	15.0	24
115	medium / medium / low / high / medium / medium	0.050	7.7	14
116	low / high / medium / high / low / medium	0.012	1.5	5
117	medium / high / medium / high / low / medium	0.005	1.8	5
118	low / low / high / medium / medium / low	0.000	1.9	5

Rule	Antecedent (Age / Education / GDS-15 / Gait speed / Physical frailty / Social frailty)	Consequent (MCI risk)	Rule weight	Support, n
119	low / medium / medium / high / low / low	0.000	9.7	17
120	medium / medium / low / medium / low / high	0.000	3.7	5
121	medium / medium / low / high / medium / low	0.000	3.9	6
122	low / medium / low / high / medium / medium	0.000	4.6	9
123	low / medium / medium / low / medium / medium	0.000	2.3	5
124	low / high / low / medium / low / medium	0.000	1.4	7
125	low / medium / high / medium / low / high	0.000	1.5	5
126	low / high / medium / medium / low / medium	0.000	2.3	5
127	low / high / medium / medium / medium / medium	0.000	3.0	7
128	low / medium / medium / high / medium / high	0.000	3.5	5
129	medium / high / medium / high / medium / low	0.000	3.5	6
130	medium / high / low / high / medium / medium	0.000	2.8	5
131	medium / high / low / medium / low / low	0.000	5.6	11
132	medium / medium / high / high / medium / low	0.000	3.8	8
133	medium / high / low / medium / medium / low	0.000	2.5	5
134	high / medium / medium / high / low / high	0.000	2.1	5
135	high / high / low / medium / medium / medium	0.000	2.5	5

Table S4 footnote. Test-set performance: ROC AUC 0.657, PR-AUC 0.442. Partitions with four and five fuzzy sets per variable yielded 111 rules (AUC 0.625) and 71 rules (AUC 0.614) respectively.

Table S5. Variance inflation factors for all candidate predictors, with a summary of the pairwise Spearman correlations.

Variable	VIF
Height (cm)	2.95
Grip strength (kg)	2.78
Female sex	2.00
Body weight (kg)	1.99
Kihon Checklist total score	1.90
Living alone	1.87
Social frailty status	1.67
GDS-15 score	1.51
Physical frailty status	1.50
Light exercise habit (frequency)	1.49
Number of household members	1.48
Regular exercise habit (frequency)	1.43
Number of medications per day	1.42
Age (years)	1.38

Variable	VIF
Usual gait speed (m/s)	1.35
Reduced daily conversation	1.28
Smoking status	1.26
Osteoporosis	1.22
Hypertension	1.22
Self-reported memory complaint	1.18
Diabetes mellitus	1.18
Years of education	1.18
Cardiac disease	1.17
Hyperlipidaemia	1.14
Knee osteoarthritis	1.13
Respiratory disease	1.11
Fracture after age 60	1.09
>=5% weight loss in 2 years	1.08
Family history of dementia	1.06
Rheumatoid arthritis / collagen disease	1.06
Malignant neoplasm	1.05
Stroke (self-reported)	1.02
Physician-diagnosed depression	1.01
Parkinson's disease	1.01

Table S5 footnote. Maximum VIF was 2.95; no predictor reached the conventional thresholds of 5 or 10. Among all 561 predictor pairs, one exceeded an absolute Spearman correlation of 0.7 (number of household members and living alone, rho = 0.731) and seven exceeded 0.5, the next largest being height and grip strength (rho = 0.689), height and female sex (rho = 0.679), body weight and grip strength (rho = 0.518), and GDS-15 score and Kihon Checklist total score (rho = 0.506).
